# Metabolic gene expression and epigenetic effects of the ketone body β-hydroxybutyrate on H3K9ac in bovine cells, oocytes and embryos

**DOI:** 10.1038/s41598-018-31822-7

**Published:** 2018-09-13

**Authors:** Juliano Rodrigues Sangalli, Rafael Vilar Sampaio, Maite del Collado, Juliano Coelho da Silveira, Tiago Henrique Camara De Bem, Felipe Perecin, Lawrence Charles Smith, Flávio Vieira Meirelles

**Affiliations:** 1 University of Sao Paulo, Department of Veterinary Medicine, Faculty of Animal Science and Food Engineering, Pirassununga, Sao Paulo postcode: 13635-900 Brazil; 2 Université de Montréal, Faculté de médecine vétérinaire, Centre de recherche en reproduction et fertilité, St. Hyacinthe, Québec, postcode: H3T 1J4 Canada

## Abstract

The rapid decline in fertility that has been occurring to high-producing dairy cows in the past 50 years seems to be associated with metabolic disturbances such as ketosis, supporting the need for research to improve our understanding of the relations among the diet, metabolism and embryonic development. Recently, the ketone body β-hydroxybutyrate (BOHB) was demonstrated to be a potent inhibitor of histone deacetylases (HDACs). Herein, we performed a series of experiments aiming to investigate the epigenetic effects of BOHB on histone acetylation in somatic cells, cumulus-oocyte complexes (COCs) and somatic cell nuclear transfer (SCNT) embryos. Treatment with BOHB does not increase histone acetylation in cells but stimulates genes associated with ketolysis and master regulators of metabolism. We further demonstrated that maturing COCs with high levels of BOHB does not affect their maturation rate or histone acetylation but increases the expression of *PPARA* in cumulus cells. Treatment of somatic cell nuclear transfer zygotes with BOHB causes hyperacetylation, which is maintained until the blastocyst stage, causing enhanced *FOXO3A* expression and blastocyst production. Our data shed light on the epigenetic mechanisms caused by BOHB in bovine cells and embryos and provide a better understanding of the connection between nutrition and reproduction.

## Introduction

The fertility of dairy cows has declined in the past half-century^1^, and the metabolic disturbances incurred by high-yielding cows seem to contribute to the poor reproductive performance^2^. The imbalance caused by the excessive expenditure of nutrients to produce high quantities of milk combined with reduced or insufficient food intake leads to a condition known as negative energy balance (NEB), which is characterized by elevated levels of nonesterified fatty acids (NEFAs) and ketone bodies (i.e., acetone, acetoacetate and β-hydroxybutyrate) in the blood serum^3^. These molecules are known to affect the follicle and oviduct environment where the oocytes and early embryos develop, culminating in reduced fertility^4,5^. On this basis, dissecting the roles played by every molecule altered in this condition will aid understanding the relations among diet, metabolism and embryonic development.

Among the molecules resulting from NEB, the ketone body β-hydroxybutyrate (BOHB) in particular is fundamental to understanding ruminant biology. It is believed that BOHB arose ~2–3 billion years ago as a molecule to store energy^6^. It works as a supplier of energy from the liver to the peripheral tissues and increases considerably during fasting, prolonged exercise and some disease states (e.g., diabetic ketoacidosis in humans and ketosis in cattle)^7^. In contrast to the situation in humans, whose circulating BOHB levels are in the micromolar range (~100–250 μM)^8^, ketone utilization is a normal part of cellular metabolism in dairy cattle, and BOHB circulating levels are in the range of 0.5–1 millimolar^9^.

Previously considered a “metabolic poison”^6^, BOHB has recently been shown to play crucial roles in cell signalling events^10^. Among these, it acts as a ligand for cell-surface receptors causing the reduction of adipose lipolysis and the release of free fatty acids (i.e., NEFAs), exerting a protective role. Furthermore, several metabolites are altered by BOHB metabolism, such as acetyl-CoA, succinyl-CoA, and NAD+, which regulate cellular metabolism^11^. An understanding of these critical and diverse signalling functions might give clues leading to better management of common disorders in dairy cows, such as ketosis and reproductive failure^12^. Surprisingly, BOHB is also a potent inhibitor of histone deacetylases (HDACs) both *in vitro* and *in vivo*^13^, further supporting its potential role in the regulation of gene expression via chromatin modifications^14^.

BOHB was shown to suppress the oxidative stress in mice by stimulating genes responsible for scavenging free radicals^13^. Since oxidative stress damages DNA and affects embryo development *in vitro*, we reasoned that exposure of cumulus-oocyte complexes or early-stage embryos to BOHB could benefit development. Moreover, since the oocytes/embryos are exposed to BOHB during their development, *in vitro* exposure may provide mechanistic insights about the aetiology of diminished fertility^2^.

Although the ketone body BOHB has been extensively studied in cows in the context of metabolism^8^, studies addressing its novel epigenetic roles are currently lacking^10^. On this basis, we performed a series of multipronged, interconnected experiments to (1) evaluate the epigenetic effects of BOHB on histone acetylation levels in somatic cells, cumulus-oocyte complexes, early cloned zygotes and blastocysts; (2) analyse genes related to the epigenome, metabolism and oxidative stress response in fibroblasts and cumulus cells; and (3) analyse the effects of HDAC inhibition with BOHB on the preimplantation developmental rates of cloned embryos, the process of histone acetylation, and the expression of important genes related to the epigenome, metabolism and oxidative stress response.

## Results and Discussion

### Effect of BOHB on Bovine Somatic Cells

#### BOHB is unable to increase H3K9ac levels in bovine somatic cells

The ketone body β-hydroxybutyrate (BOHB), canonically known for its role as a supplier of energy during states of privation^6^, was discovered to be an epigenetic modulator as well. Mice infused with BOHB showed increased histone acetylation in the kidney. Additionally, HEK293 human embryonic kidney cells treated with BOHB in culture medium for 8 h increased their H3K9ac levels in a dose-dependent manner. BOHB inhibited class I and class II HDACs with a median inhibitory concentration (IC_50_) ranging from ~2 to 5.3 mM. These data indicate that BOHB is a potent endogenous HDAC inhibitor both *in vivo* and *in vitro*^13^.

Curiously, in contrast to their role in humans, ketone bodies are a normal energy source throughout the adult life of a ruminant^9^. However, bovines are sensitive to elevation of BOHB levels in the bloodstream. Dairy cows with BOHB levels above 1.4 mM are considered to be in ketosis^15^. Epidemiological studies in dairy herds have demonstrated that cows with BOHB levels ~2 mM present reduced milk production^15^. In the case of persistent nutritional imbalance in lactating dairy cows, they can develop severe clinical ketosis (BOHB > 3 mM)^16^, where the BOHB levels can reach ~6 mM. On this basis, we selected two relevant concentrations of BOHB (2 and 6 mM) in this experiment to investigate whether BOHB affects histone acetylation in bovines.

**Figure 1:**
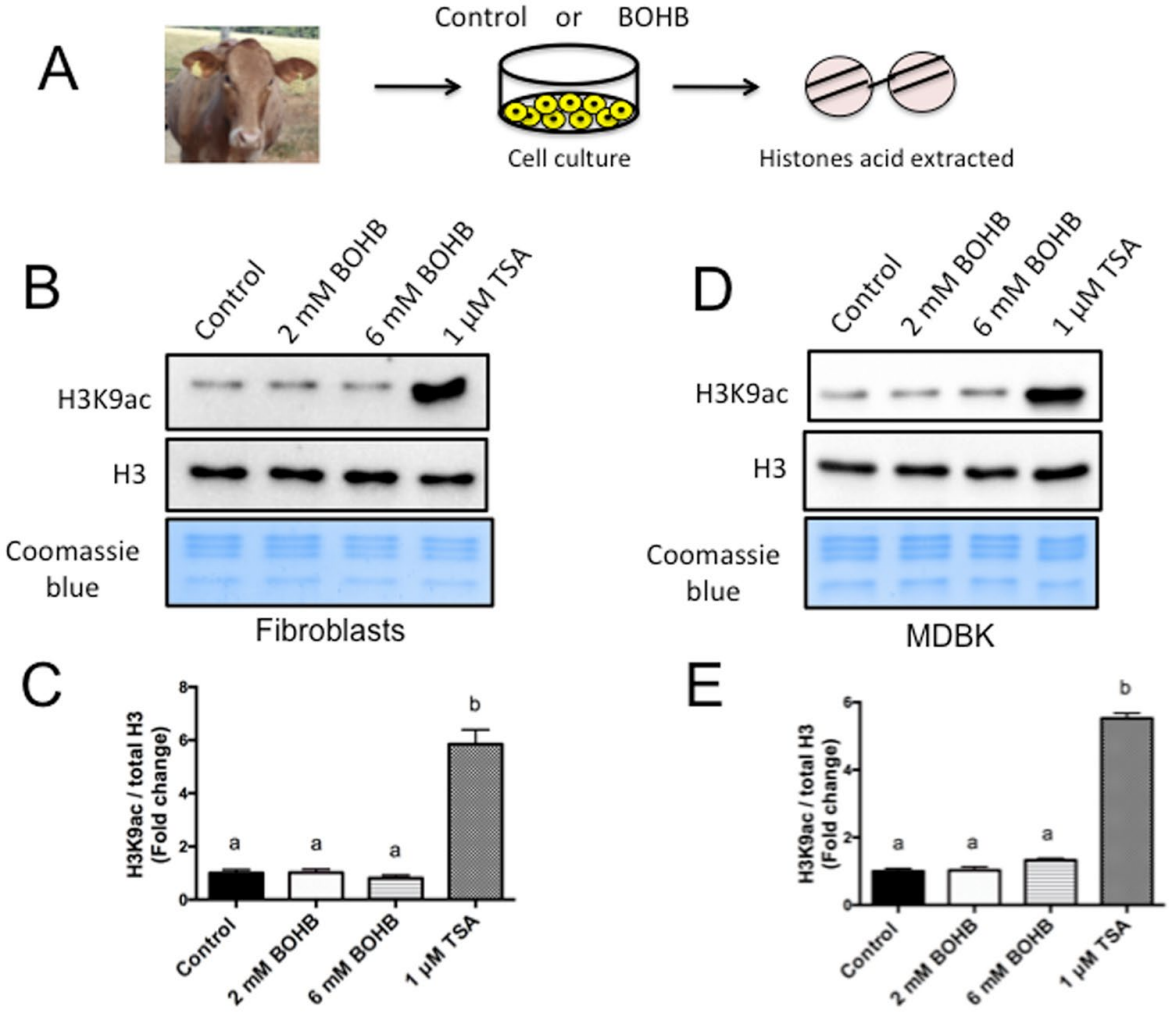
Short-term exposure to β-hydroxybutyrate (BOHB) does not increases H3K9ac levels in bovine cells. (**A**) Diagram illustrating the isolation of bovine fibroblasts, treatment during cell culture, and subsequent acid extraction of histones for western blot analysis. (**B**) Immunoblots and (**C**) quantification values of H3K9ac in skin fibroblasts treated for 8 h with 0 mM (control), 2 mM, or 6 mM BOHB or 1 μM TSA. (**D**) Immunoblots and (**E**) quantification of H3K9ac in MDBK cells treated for 8 h with 0 mM (control), 2 mM, or 6 mM BOHB or 1 μM TSA. Data are representative of 3 independent WBs, and H3K9ac levels were calculated in relation to total histone 3 (H3). Data are presented as fold change in relation to the control group and are shown as the mean ± s.e.m. Different letters indicate a significant difference (P < 0.05). The western blot images were cropped for illustrative purposes. The full gels/blots are presented in Supplemental Fig. 3.

To determine whether BOHB possess HDAC inhibitory activity in bovine somatic cells, we treated bovine primary skin fibroblasts and Madin-Darby bovine kidney (MDBK) cells for 8 h with 0, 2 and 6 mM of BOHB, as well as 1 μM of the potent HDAC inhibitor trichostatin A (TSA) as a positive control (Fig. 1A). Following histone acid extraction, measures of H3K9ac, a robust and well-characterized post-translational modification associated with gene expression, did not indicate an increase in histone acetylation levels in either cell type, regardless of the dose of BOHB utilized (Fig. 1B–E). As expected, treatment with TSA greatly increased the H3K9ac levels of fibroblasts (~5.85-fold) and MDBK cells (~5.52-fold) relative to untreated cells (0 mM BOHB; Fig. 1B–E). We further tested another concentration (4 mM) and another molecule closely related to BOHB, the canonical HDAC inhibitor sodium butyrate (NaBu). We observed that NaBu and TSA dramatically increased the levels of H3K9ac, whereas BOHB did not affect the H3K9ac levels in any dose tested (2, 4 and 6 mM – Supplemental Fig. 1).

**Figure 2:**
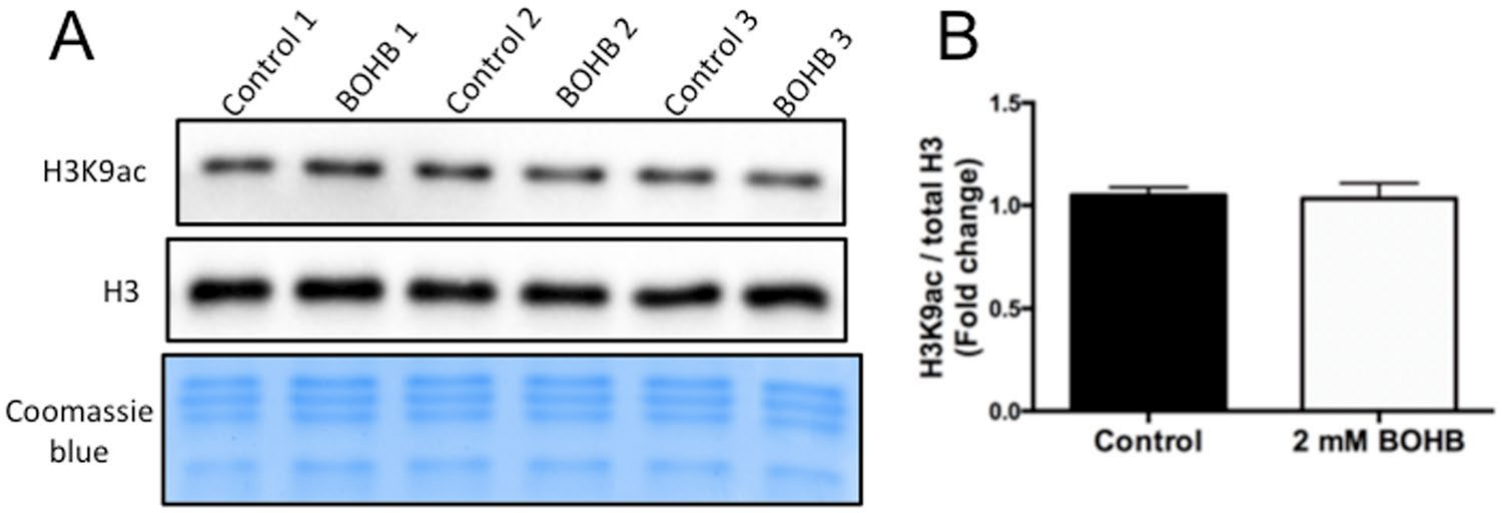
Prolonged exposure to β-hydroxybutyrate (BOHB) does not increase H3K9ac levels in bovine fibroblasts. (**A**) Immunoblots for H3K9ac of skin fibroblasts that were either untreated (controls) or treated with BOHB (2 mM) for 96 h. (**B**) Quantification of H3K9ac levels in fibroblasts treated for 96 h. Data are representative of three pairs of biological replicates. The H3K9ac levels were calculated in relation to total histone 3 (H3). Data are presented as fold change in relation to the control group, and the values are shown as the mean ± s.e.m. Different letters indicates significant difference (P < 0.05). The western blot images were cropped for illustrative purposes. The full gels/blots are presented in Supplemental Fig. 4.

Since cows with ketosis usually present elevated BOHB serum levels (~2 mM) and reduced glucose levels for several days^16^, we decided to examine whether histone acetylation levels would increase with exposure of bovine cells for a longer period (96 h) in a culture medium with 2 mM BOHB and 2.2 mM of glucose, the lowest reference levels for bovine blood^17^. As in the shorter (8 h) exposure period, H3K9ac levels were not increased by prolonged exposure to 2 mM BOHB, indicating that acetylation levels were unaffected in bovine fibroblasts even after 4 days of exposure (Fig. 2A and B), while TSA caused an ~4-fold increase relative to untreated controls (Supplemental Fig. 2).

In agreement with our results, pioneering experiments also failed to demonstrate hyperacetylation in cells treated with β-hydroxybutyrate^18^. Additionally, our data are in accordance with the findings of Xie *et al*., in which HEK293 cells showed no increase in histone acetylation in response to BOHB^19^. On the other hand, these authors identified a new type of histone mark, lysine β-hydroxybutyrylation (Kbhb), that is dramatically induced in response to elevated β-hydroxybutyrate levels in cultured cells and in the livers of mice subjected to prolonged fasting or streptozotocin-induced diabetic ketoacidosis^19^. Their findings identify histone β-hydroxybutyrylation as a novel mechanism by which ketone bodies regulate cellular physiology, suggesting that the utilization of ketone bodies by the cells alters gene expression through metabolite-directed histone modifications^10,19^. This new type of histone modification warrants further, in-depth investigation in bovines.

#### Medium acidification reduces the global level of histone acetylation

The ruminal microbiome converts cellulose and starch to volatile fatty acids (e.g., acetic acid, propionic acid, and butyric acid), and ruminal epithelial cells metabolize these short-chain fatty acids and generate large amounts of D-β-hydroxybutyric acid, acetoacetic acid, and lactic acid^9^. In order not to disturb intracellular pH (pHi) and thus disrupt cell physiology, these acids must be extruded from the cytosol^20,21^. Therefore, ketone bodies are not only involved in energy metabolism but also potentially associated with other cellular responses, such as the interconversion of D-β-hydroxybutyrate and acetoacetate as a means of controlling pHi^8^.

Recently, studies have shown that, as pHi decreases, histones are globally deacetylated by histone deacetylases (HDACs), indicating that histone acetylation regulates intracellular pH^22^. Additionally, cells release acetate anions and protons using monocarboxylate transporters (MCTs) to regulate the intracellular pH and prevent further acidification^22^. For instance, MCT1 is expressed in the stratum basale of the ruminal epithelium and may function as a main mechanism for removing ketone bodies and lactate, together with H^+^, from the cytosol into the bloodstream^21^.

Interestingly, BOHB is chemically a carboxylic acid, and it is released from the cells by these monocarboxylate transporters^7^. We noticed that the supplementation of cell culture medium with BOHB slightly decreased the pH of the medium due to the acidic nature of BOHB; therefore, we adjusted the pH of the culture media with NaOH before adding the cells.

Changes in extracellular pH (pHe) are known to induce corresponding changes in pHi. Acidification of the cell culture medium (dropping the pH to 6.5) was shown to significantly reduce histone acetylation in cells^22^. Indeed, the extracellular addition of 20 mM D-β-hydroxybutyrate, acetoacetate, or lactate (20 mM) resulted in intracellular acidification of cultured ruminal epithelial cells^21^. We reasoned that *in vivo*, when ketone levels rise or the extracellular milieu in the rumen turns acidic, the ruminal epithelial cells might decrease their histone acetylation as a mechanism to maintain the pHi; thus, we decided to investigate whether the acidification of the culture media could affect the global levels of histone acetylation. The association between cellular acidification and acetylation could explain how, when the extracellular milieu becomes acidic in certain pathological situations, (i.e., ruminal pH decreases in subacute ruminal acidosis (SARA)), the environment can affect gene expression, leading to a collapse in the energy production system and disruption of the ruminal epithelial barrier^20^.

**Figure 3:**
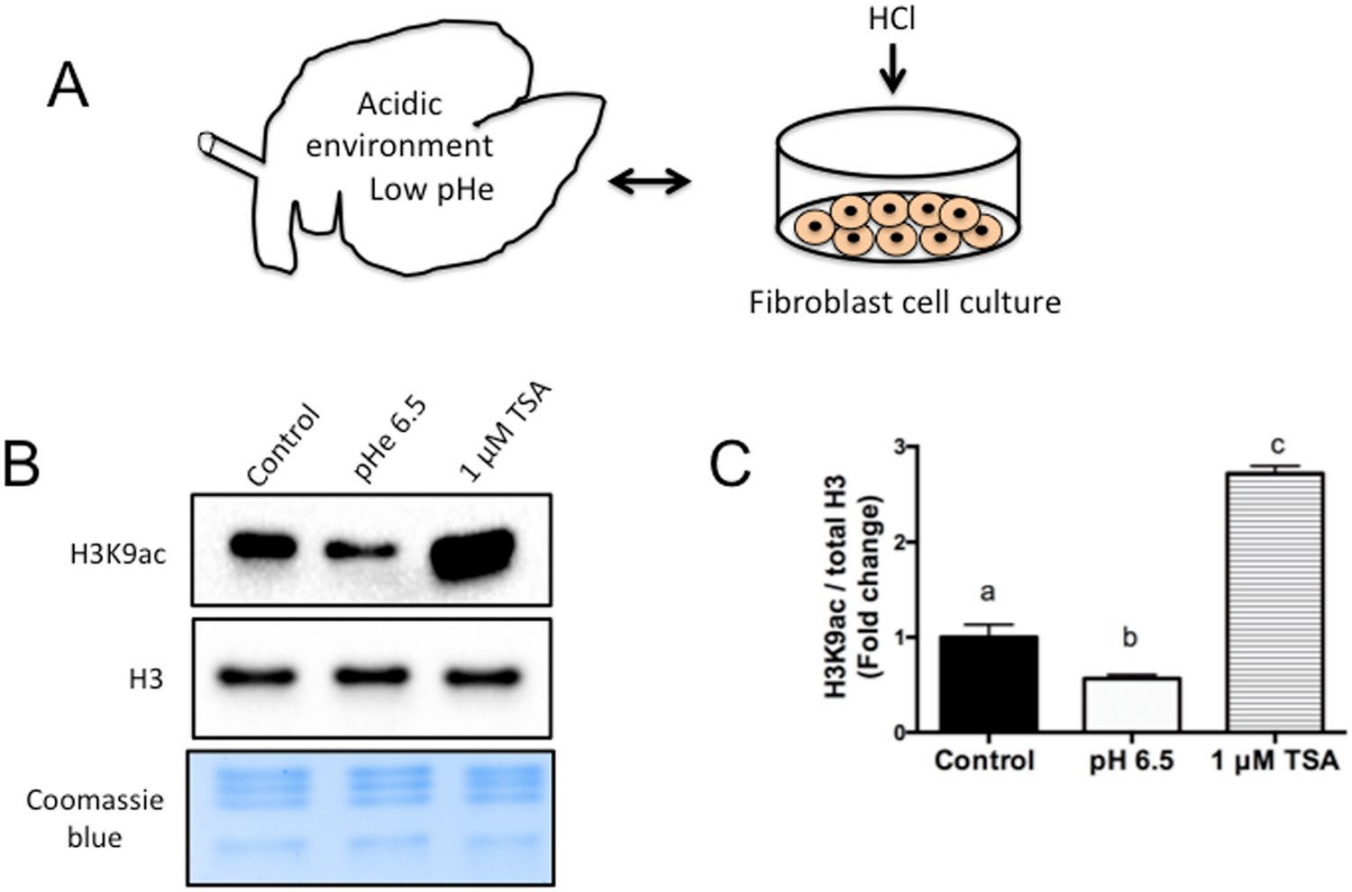
Acidification of the cell culture medium reduces the global level of histone acetylation. (**A**) Diagram illustrating a bovine rumen with low pHe and a petri dish containing cell culture medium acidified by addition of HCl. (**B**) Immunoblots for H3K9ac from fibroblasts cultured in an acidic environment for 4 h. (**C**) Quantification of H3K9ac levels in fibroblasts from the control group, acidified with HCl or cultured with 1 µM TSA (positive control). The H3K9ac levels were calculated in relation to total histone H3 (H3). Data are presented as fold change in relation to the control group, and values are shown as the mean ± s.e.m. Different letters indicate a significant difference (P < 0.05). The western blot images were cropped for illustrative purposes. The full gels/blots are presented in Supplemental Fig. 5.

Since β-hydroxybutyrate is a strong organic acid, we reasoned that the acidic nature of BOHB may have contributed to the lack of observed histone hyperacetylation in the experiments above. To test the hypothesis (Fig. 3A) that a pH drop in the extracellular milieu – which, *in vivo*, may be caused by ruminal acids (e.g., propionate, butyrate, acetate, β-hydroxybutyrate) – reduces global levels of histone acetylation, we utilized an *in vitro* model of cell culture medium acidification^22,23^. We acidified fibroblast cell culture medium (pHe 6.5) by addition of HCl^23^, acid-extracted the histones and measured the H3K9ac levels by western blot (Fig. 3B). We observed that the reduction of the pHe to 6.5 led to a decrease of ~1.8-fold in the level of H3K9ac (Fig. 3C).

This result indicates that an acidic extracellular environment, similar to the one caused *in vivo* by ingestion of highly fermentable carbohydrates or elevated levels of BOHB^9,20^, causes a significant reduction in the global level of histone acetylation. In addition, these observations suggest that, *in vivo*, the cells excrete ketones to manage stress and maintain pHi, thus counterbalancing the hyperacetylation caused by BOHB inhibiting HDAC^21,22^. Although outside the scope of this manuscript, further investigation is warranted to elucidate such mechanisms.

#### BOHB increases the expression of genes related to ketolytic pathways and caloric restriction responses

Recent findings support a model in which BOHB may be the key molecule mediating the beneficial effects caused by caloric restriction^24^. For instance, BOHB might function as a link between the environment, in this case the diet of the animal, and the regulation of gene expression via chromatin modifications^10^. The main physiological response of animals suffering from NEB is to maintain homeostasis by mobilizing body fat (and protein) reserves. This response often fails in high-production dairy cattle, leading to the accumulation of high concentrations of ketone bodies in the blood, i.e., ketosis^25^. NEB and ketosis are potentially detrimental to the health of the animals and have a significant negative economic impact on the dairy cattle industry, which further justifies the need to understand NEB/ketosis at the molecular level^26^. The analysis of genes important for antioxidative responses, master regulators of metabolism, caloric restriction and epigenetic processes may shed light on the mechanisms utilized by dairy cows to cope with such adverse conditions and maintain homeostasis.

**Figure 4:**
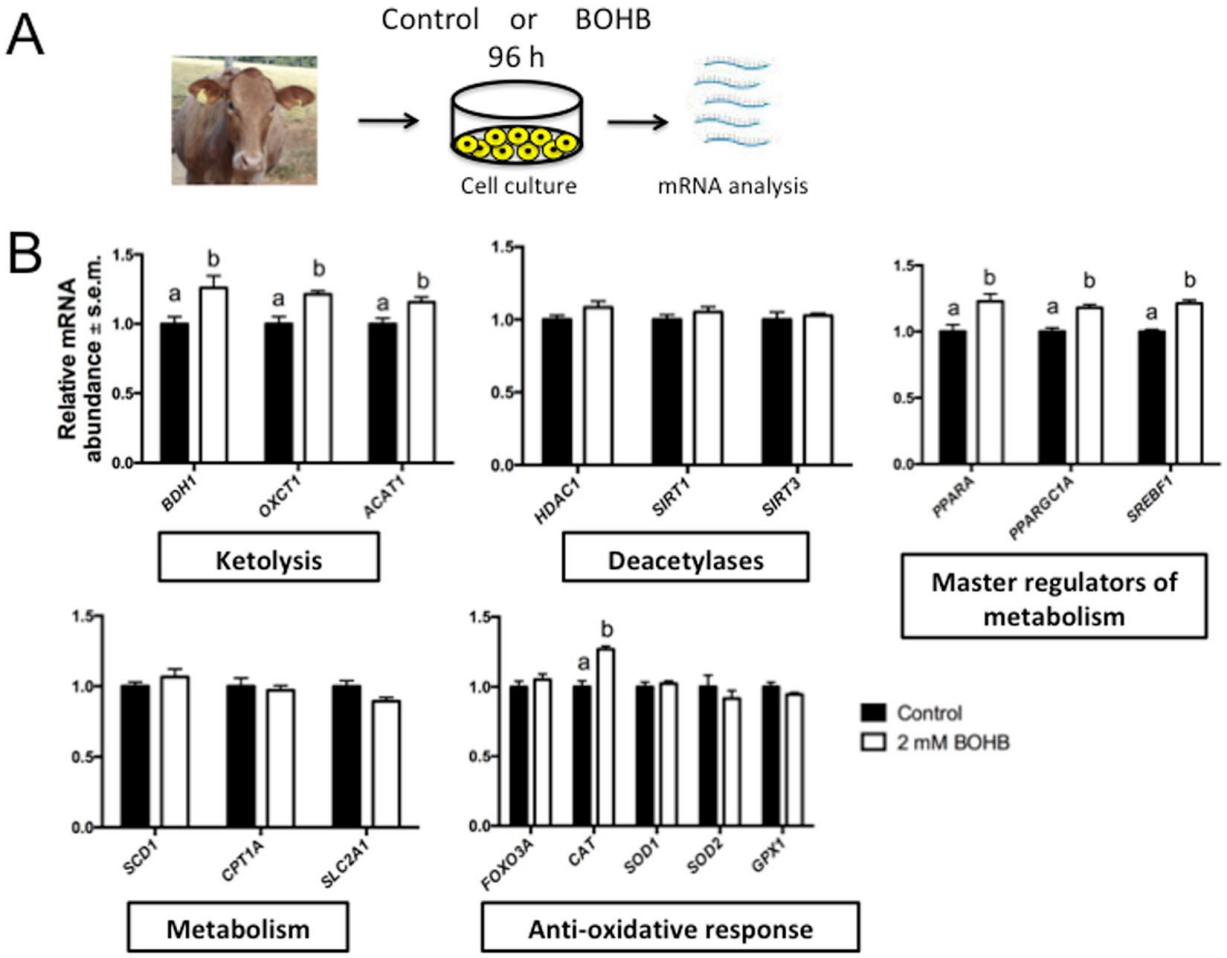
β-Hydroxybutyrate (BOHB) increased the expression of genes encoding enzymes related to ketolytic pathways and genes that phenocopy caloric restriction responses. (**A**) Diagram illustrating the treatment of bovine skin fibroblasts for 96 h with 2 mM of BOHB to mimic levels attained systemically by ketotic cows. (**B**) Quantification of transcripts in bovine skin fibroblasts cultured either with (2 mM) or without (control) BOHB for 96 h. Transcript quantities were normalized to the geometric mean of three housekeeping genes (*GUSB*, *PPIA* and *RPL15*). Data are presented as fold change in relation to the control group, and the values are shown as the mean ± s.e.m. Gene expression data from 5 biological replicates. Different letters indicate a significant difference (P < 0.05).

Postpartum NEB/ketosis in cows lasts for several weeks (~10–12), and *in vivo* the liver is constantly synthesizing BOHB and releasing it into the bloodstream^7,15^. Aiming to gain insights into the effects of BOHB on cellular physiology, we used bovine fibroblasts as a tractable platform to interrogate the responses of cells to BOHB treatment. To mimic an *in vivo* scenario, we decided to treat fibroblasts with 2 mM BOHB for a prolonged period (96 h). We replenished the BOHB every day, and at the end of treatment we quantified its effect on gene expression (Fig. 4A).

BOHB is the most abundant circulating ketone body. It is released from the liver to the blood and transported to the peripheral tissues for terminal oxidation. This process occurs in the mitochondria, in a set of reactions termed ketolysis^8^. After uptake by the cells, BOHB is converted to acetoacetate by 3-hydroxybutyrate dehydrogenase 1 (*BDH1*). Subsequently, it is converted to acetoacetyl-CoA by a succinyl-CoA-dependent transferase (succinyl-CoA:3-ketoacid CoA transferase, SCOT, encoded by the nuclear gene *OXCT1*), which is the key reaction that enables the utilization of ketone bodies as energy substrates. In the final step, acetoacetyl-CoA is cleaved into two molecules of acetyl-CoA by acetyl-CoA acetyltransferase 1 (*ACAT1*). These molecules are then oxidized in the TCA cycle and the respiratory chain for ATP synthesis^8^. We carried out qRT-PCR and observed upregulation of the transcripts for all of these 3 enzymes – *BDH1*, *OXCT1* and *ACAT1* (Fig. 4B). In addition to its role as an HDAC inhibitor, BOHB may promote hyperacetylation in cells by increasing the intracellular pools of acetyl-CoA^7^. However, as stated above, neither short nor prolonged exposure to BOHB causes a shift in the reaction equilibrium towards acetylation.

Sirtuins, a distinct family of NAD^+^-dependent deacetylases that modulate metabolic processes, are not known to be regulated by BOHB. However, the relative sparing of cytoplasmic NAD+ levels with utilization of BOHB rather than glucose is thought to alter the activity of metabolic enzymes such as sirtuins^10^. As we observed upregulation of enzymes related to BOHB metabolism, we decided to analyse the transcripts of sirtuin 1 (*SIRT1*) and sirtuin 3 (*SIRT3*), which localize in the nucleus and mitochondria, due to the role they play in regulating essential cell reprogramming events during nutrient deprivation and ketogenesis^27^. When we analysed the levels of those transcripts, we did not observe any difference between the BOHB-treated and control groups (Fig. 4B). Although BOHB supplementation did not increase the abundance of sirtuin transcripts, we cannot rule out the possibility that the activity of these enzymes was affected. Since BOHB increases histone acetylation and sirtuins causes deacetylation, these opposing activities warrant further investigation^7,27^.

In dairy cows, elevated levels of circulating ketone bodies are associated with an elevated percentage of fat in the milk^28^. In ruminants, BOHB is the main precursor of milk fat synthesis^29^. Since sterol regulatory element-binding transcription factor 1 (*SREBF1*) plays crucial roles in lipid synthesis, we decided to investigate whether the expression of this gene is affected by BOHB supplementation. Our results showed that BOHB treatment increased the expression of *SREBF1* (Fig. 4B). Treatment of cultured mammary epithelial cells with BOHB activates *SREBF1* and increases the secretion of triacylglycerol^28^. These cell culture models might explain at least one mechanism by which NEB increase the fat content of cows’ milk.

The bovine stearoyl-CoA desaturase (*SCD*) gene plays an important role in the bovine mammary gland, producing up to 90% of the conjugated linoleic acid in bovine milk^30^. This enzyme is under the control of *SREBF1* and has been implicated in the aetiology of postpartum lipidosis. Ketotic cows present down-regulation of *SCD* in the liver, which might impair VLDL synthesis and secretion^31^. Our cell culture model showed that the solo exposure of cells to BOHB does not affect *SCD* transcript levels (Fig. 4B), suggesting that other molecules are implicated in the metabolic disturbances during lipidosis.

The enzyme carnitine palmitoyltransferase 1A (*CPT1A*) is necessary for the transport of NEFA into the mitochondria for ketone body synthesis and has been associated with the aetiology of ketosis^8^. However, no changes in expression were observed after exposure to BOHB (Fig. 4B), which supports previous studies where no change in the expression of *CPT1A* was observed in cows with ketosis induced by food restriction^31^. These data suggest that mitochondrial capacity for long-chain fatty acid oxidation is not affected by nutrition-induced ketosis or cell treatment with BOHB.

Under conditions of NEB or fasting, ketone bodies are released into the bloodstream to serve as an additional fuel source for metabolically active tissues, such as muscle and brain. In these tissues, they are utilized for energy production, sparing glucose and promoting higher blood glucose concentrations^25^. We quantified the expression levels of the ubiquitous glucose transporter solute carrier family 2 member 1 (*SLC2A1*) to evaluate whether BOHB contributes to spare glucose in cattle. We observed a downregulation, albeit not statistically significant (p = 0.0612), of *SLC2A1* in fibroblasts treated with BOHB (Fig. 4B). In humans, infusion of BOHB reduced myocardial glucose uptake^32^. In contrast, fasting-induced production of BOHB enhances expression of the glucose transporter gene *SLC2A1* (Glut1) via H3K9ac hyperacetylation of its regulatory region in mouse brain endothelial cells^33^. These observations suggest that priority is given to the brain and that downregulation of Glut1 might serve as a mechanism to spare glucose in bovine fibroblasts^9,34^. In support of this possibility, we observed in a preliminary experiment that supplementation of culture medium with high concentrations (6 mM) of BOHB decreased the expression of the glucose transporters *SLC2A1* and *SLC2A3* (Supplemental Fig. 6).

Adaptation to fasting and nutrient deprivation (e.g., NEB in cows) requires a thorough reprogramming of the metabolism^25^. Two major players in these responses are peroxisome proliferator-activated receptor α (*PPARA*) and peroxisome proliferator-activated receptor-γ coactivator 1α (*PPARGC1A)*. *PPARA* is the master regulator for the induction of genes necessary for lipid metabolism and ketone body biosynthesis/import^35^. *PPARGC1A* is a master regulator of mitochondrial biogenesis/function and cooperates with *PPARA* to regulate ketolysis and ketone body utilization in peripheral tissues^36^. Therefore, *PPARA* and *PPARGC1A* interactions function to reprogram gene expression in response to nutritional deprivation. We observed that treatment of fibroblasts with BOHB led to an increase in the abundance of *PPARA* and *PPARGC1A* transcripts (Fig. 4B). These results demonstrated that the addition of BOHB to the culture medium signals cells to stimulate the master regulators of the “fasting” response. This effect of BOHB probably reflects an evolutionary response that helps cells reprogram their metabolism, cope with the nutritional stress and reroute their physiological processes to use alternative energy sources during states of poor food supply^24,34^.

In addition to its metabolic effects, BOHB was recently described to be an endogenous epigenetic modifier^13^. Modulating HDAC activity by pharmacological means regulates lifespan in model organisms^37^. We did not observe effects on histone deacetylase 1 (*HDAC1*) transcript abundance in response to BOHB (Fig. 4B). The kinetics of BOHB inhibition of HDACs suggests that it is a competitive inhibitor that acts by chelating zinc in a similar way to butyrate^7,10^ and, based on our finding, not by regulating HDAC1 at the transcriptional level. Another striking feature of HDAC inhibition is the induction of resistance to multiple forms of cellular stress. BOHB has beneficial metabolic and cytoprotective effects, which may be due to induction of antioxidant genes^7,13,38,39^. We investigated whether BOHB treatment increases the abundance of forkhead box O3 (*FOXO3A*), superoxide dismutase 1 (*SOD1*) and 2 (*SOD2*), catalase (*CAT*) and glutathione peroxidase 1 (*GPX1*) transcripts. Except for the upregulation of *CAT* transcripts, the stress resistance genes that we analysed did not respond to BOHB (Fig. 4B). Mice infused with BOHB show marked increases in Foxo3a and two of its well-defined targets, Sod2 and catalase^13^. Our data suggest that BOHB can enhance at least one antioxidant gene *in vitro*. This might partially explain the broad pro-survival effect that BOHB exerts on *in vitro* models of cellular stress^8,39^.

### Effect of BOHB on cumulus-oocyte complexes

#### Exposure of cumulus cells to β-hydroxybutyrate during *in vitro* maturation does not alter acetylation levels but upregulates the “fasting” response gene PPARA

Cumulus cells play several critical roles during oocyte development and maturation, including the exchange of molecules such as ions, metabolites, amino acids and growth factors that promote meiotic resumption, protection against oxidative stress, and cytoplasmic maturation of the oocytes^40^. Although the oocyte is transcriptionally inactive during the maturation process, recent findings have demonstrated that cumulus cells can supply the oocyte with entire mRNAs and long non-coding RNA molecules^41,42^.

**Figure 5:**
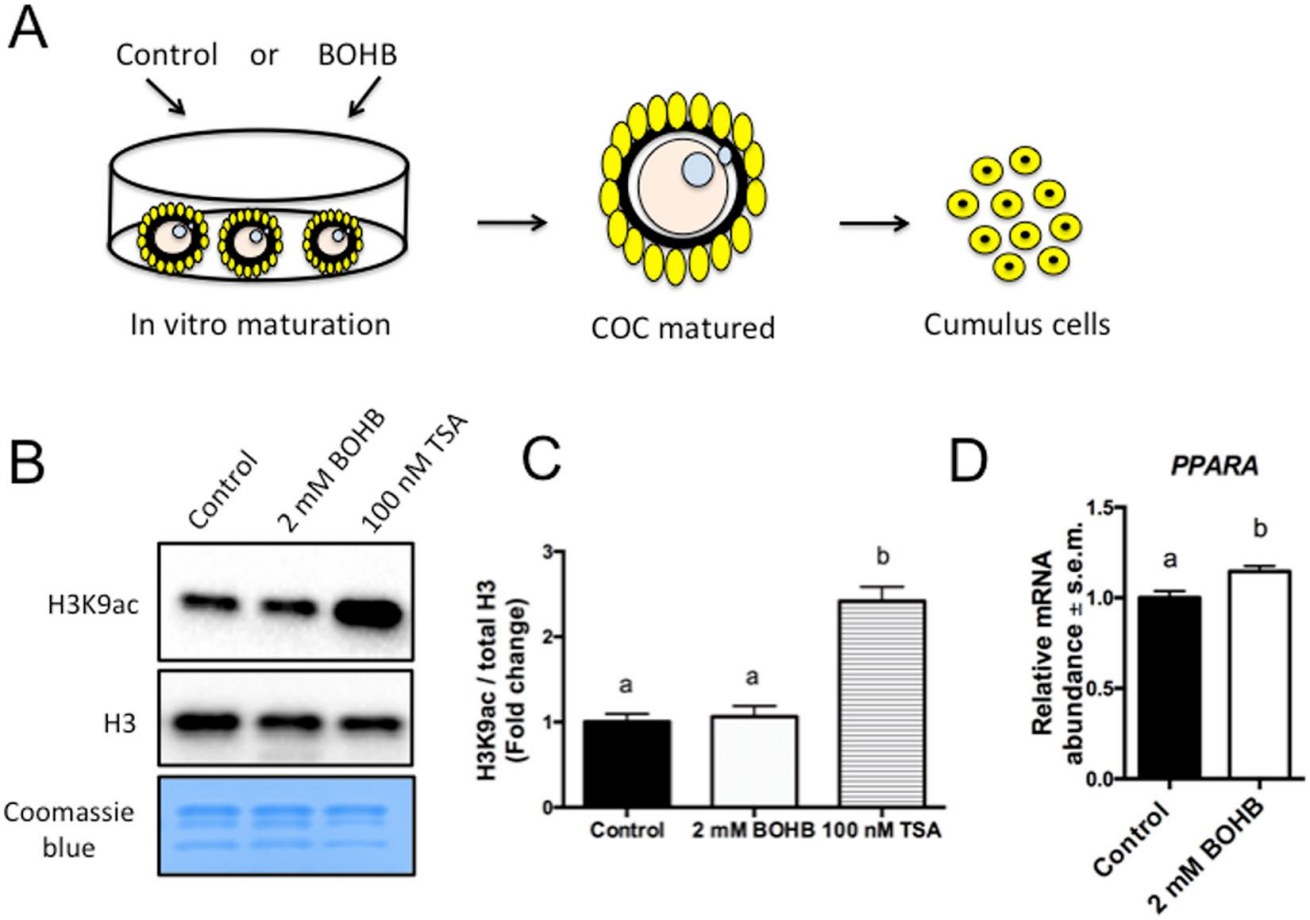
Exposure of cumulus cells to β-hydroxybutyrate (BOHB) does not alter acetylation levels but upregulate the “fasting” response gene PPARA. (**A**) Diagram illustrating the exposure of cumulus-oocyte complexes (COCs) to BOHB. After BOHB treatment during *in vitro* maturation, the oocytes were stripped off and the cumulus cells were used for western blots and gene expression analysis. (**B**) Immunoblots for H3K9ac, total histone H3 (H3) and histones stained with Coomassie Blue. (**C**) Quantification of H3K9ac levels in cumulus cells from COCs maturated alone (negative control) or in the presence of BOHB (2 mM) and TSA (100 nM; positive control) for 21–23 h. H3K9ac levels were calculated in relation to total H3. (**D**) Relative mRNA abundance of *PPARA* in control and BOHB-treated cumulus cells. Data are presented as fold change in relation to the control group, and values are shown as the mean ± s.e.m. Gene expression data from 6 biological replicates. Different letters indicate a significant difference with respect to the control group (P < 0.05). The western blot images were cropped for illustrative purposes. The full gels/blots are presented in Supplemental Fig. 7.

Pioneer experiments have demonstrated that some metabolic changes that occur in the blood of early postpartum high-yielding dairy cows are reflected in the follicular fluid^5^. Remarkably, there is a strong correlation between levels of the ketone body BOHB in the serum and in the intrafollicular fluid, the latter of which is the microenvironment where the cumulus-oocyte complexes (COCs) are developing/maturing^43^. That said, in ketotic cows (BOHB serum levels ~2 mM), the oocyte’s companion cells, the cumulus cells, are exposed to high levels of this endogenous metabolite. To mimic this process *in vitro*^43^, we exposed COCs to 2 mM of BOHB during *in vitro* maturation (Fig. 5A) to investigate whether the treatment would affect H3K9ac levels. Additionally, since histone acetylation is associated with gene transcription, we decided to verify whether the expression of metabolically important genes in cumulus cells, which may affect the oocyte maturation/development, are altered. Although TSA treatment increased the acetylation level ~2.42-fold, we found that H3K9ac levels did not differ between the BOHB and control groups (Fig. 5B,C). Next, we analysed the same genes evaluated in fibroblasts (see Supplemental Fig. 8) and showed that only *PPARA* was overexpressed in response to BOHB exposure (Fig. 5D).

*PPARA* is a master regulator of lipid metabolism, and its expression is strongly induced during fasting and caloric restriction^35^. This supports the view that BOHB may also act as a signalling molecule within the ovarian follicle, stimulating the expression of specific genes within the cumulus cells to help the oocyte cope with an adverse situation. In neurons, the main mechanisms mediating the neuroprotective effects of the *PPARA* transcription complex appear to reroute metabolism away from glucose metabolism and towards alternative substrates^36^. Because cows in ketosis are normally undergoing massive lipolysis to provide energy to the organism, an upregulation of *PPARA* may, overall, stimulate lipid metabolism and reprogram these cells to use alternative sources of energy.

#### Oocyte maturation under β-hydroxybutyrate (BOHB) exposure does not affect the histone acetylation levels or maturation rates

**Figure 6:**
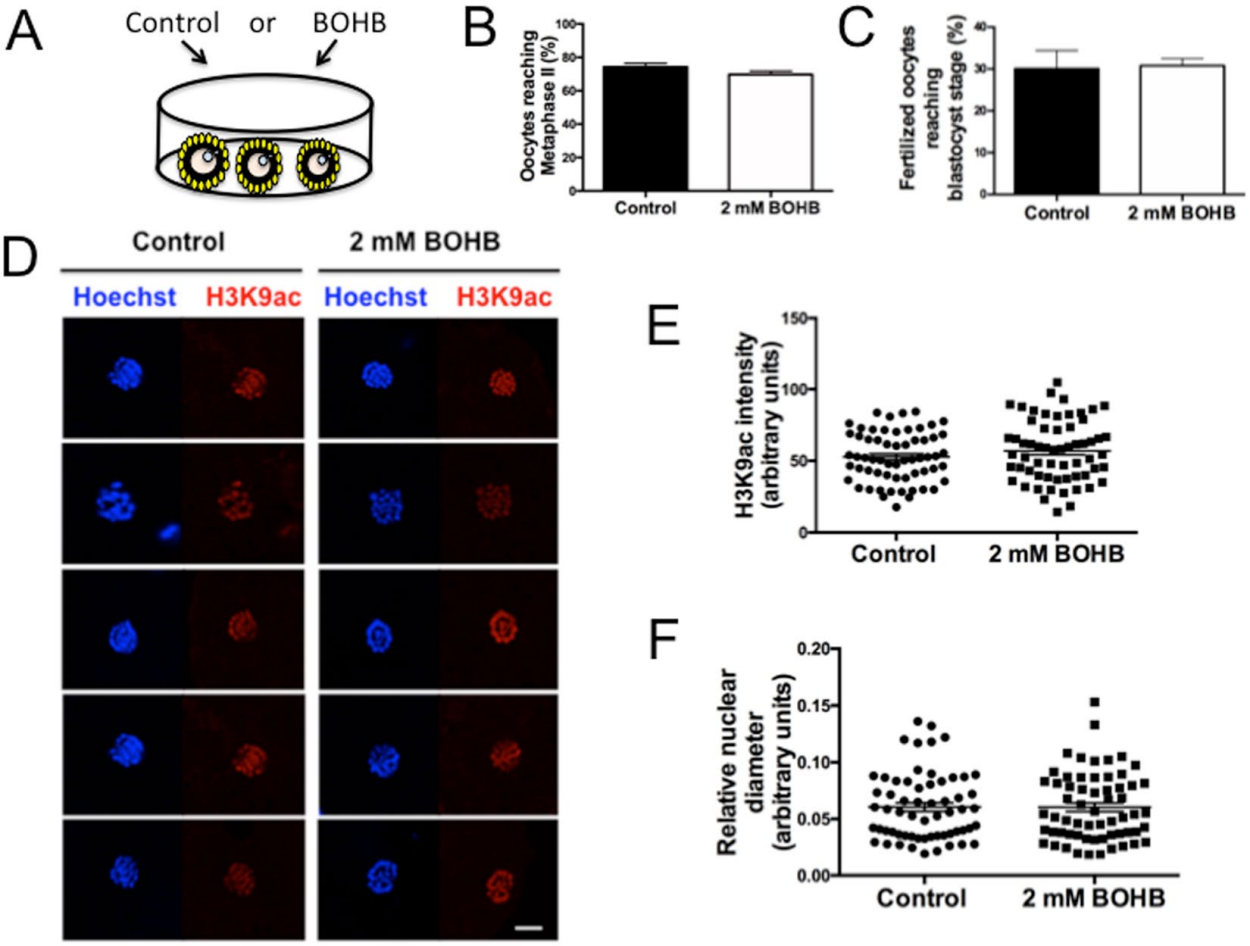
Oocyte maturation in the presence of β-hydroxybutyrate (BOHB) does not affect the histone acetylation levels or maturation rates. (**A**) Diagram illustrating oocytes maturing *in vitro* in media supplemented or not supplemented with 2 mM of BOHB. (**B**) Maturation rate of oocytes *in vitro* matured in control medium (0 mM BOHB) or medium with 2 mM BOHB. (**C**) Blastocyst rate after fertilization of oocytes matured with or without 2 mM BOHB. (**D**) Immunocytochemical detection of H3K9ac (red) and DNA (blue) in maturated oocytes. Panel of 5 representative images per group (from a total of 61). All images were taken at the same magnification (63×). (**E**) Quantification of H3K9ac intensity in the metaphase spindle of oocytes shown in panel C. (**F**) Measurement of the relative area of the metaphase plate of the oocytes shown in panel C. Data are expressed as the mean ± s.e.m. (P > 0.05). The scale bar corresponds to 10 μm.

Modern high-producing dairy cows present reduced fertility, which might be caused by altered levels of metabolites (e.g., NEFAs and ketone bodies) originating from metabolic disorders that affect the maturing gametes^2^. In the past few decades, evidence has accumulated that oocytes maturing in this altered microenvironment have diminished quality and capacity for embryo development^43^. Circulating BOHB levels are remarkably similar between the serum and the follicular fluid^5^. On this basis, we decided to utilize an *in vitro* maturation model^44^ based on *in vivo* observations^5,43^ to gain mechanistic insights about the aetiology of diminished fertility. We exposed immature cumulus-oocyte complexes (COCs) to 2 mM BOHB (Fig. 6A), a level found in the circulating blood and follicular fluid of ketotic cows. Subsequently, we evaluated whether this BOHB exposure would affect the capacity of the oocyte to reach metaphase II and to develop to the blastocyst stage after fertilization. Additionally, we measured the effects of BOHB exposure on H3K9ac levels in metaphase II oocytes. COCs were cultured in the presence of 2 mM BOHB during the IVM (~21–23 h), and the denuded oocytes were assessed for meiotic resumption based on the extrusion of the first polar body. Using 10 independent replicates, we cultured oocytes in the presence (n = 1013) or absence (n = 994) of 2 mM BOHB and found no significant difference in maturation rates between the treated and control groups (69.74 ± 2.01 vs. 74.27 ± 2.22%, P = 0.136; Fig. 6B), indicating that the supplementation of IVM medium with high levels of BOHB does not affect the ability of oocytes to undergo meiotic resumption *in vitro*. Further, we fertilized BOHB-treated and untreated COCs to evaluate whether maturation under high levels of BOHB has a carry-over effect on the oocytes’ capacity to produce blastocysts^12^. After 3 independent replicates, fertilized oocytes previously matured in the presence (n = 187) and absence (n = 189) of 2 mM BOHB presented similar cleavage rates (control 81.84 ± 3.40 vs BOHB 77.16 ± 10.72; P = 0.40) and blastocyst production (control 30.07 ± 4.36 vs BOHB 30.80 ± 1.69; P = 0.68) rates (Fig. 6C), indicating that *in vitro* oocyte maturation under elevated levels of BOHB does not compromise the early development of embryos derived from those oocytes.

In agreement with our data, Leroy *et al*. did not observe any adverse effect of BOHB supplementation on the developmental capacity of oocytes^44^. Nevertheless, those authors observed an adverse effect when the glucose levels were also reduced in the maturation medium concomitantly with the BOHB supplementation^44^, suggesting that all molecules altered by NEB might work together to create an adverse biochemical microenvironment inside the follicle, culminating in poor gamete/embryo development^43^.

The absence of a negative effect in our system also might be due to the short period of exposure to BOHB. Ketosis is a complex condition in which several molecules are altered^9^ and is almost impossible to mimic *in vitro*^43^. Additionally, because the disordered metabolic conditions last several days or even weeks *in vivo*^15^, the cumulative effect of high levels of BOHB on the growing follicle/oocyte might be necessary to adversely affect developmental competence^43^. In contrast to mice^45^, no suitable *in vitro* protocol is available for the culture of bovine follicles for long periods that would simulate the growth of follicles/oocytes under these adverse conditions. Nevertheless, the short-term bovine exposure model used in this study and elsewhere^44,46^ allows investigation of the mechanisms through which oocytes are affected by exposure to a single molecule during the final stage of maturation. From our data, we can infer that BOHB might be a protective molecule that helps the organism – or, in this specific case, the cumulus-oocyte complex – cope with the altered environment, playing diverse roles such as sparing glucose, scavenging free radicals, providing energy and stimulating genes involved in lipid metabolism^10,11^.

Supporting the above view, Van Hoeck *et al*. showed that the addition of NEFAs to maturation medium is sufficient to affect oocyte developmental competence, demonstrating that high levels of these fatty acids are indeed toxic, even if they do not affect other molecules in IVM media (i.e., glucose, ketone bodies, and amino acids)^46^. By contrast, elevated levels of BOHB, as used herein with cumulus cells and skin fibroblasts, triggered responses by genes responsible for reprogramming metabolism and coping with stress (**↑***CAT*, **↑***PPARA*, **↑***PPARGC1A*).

The process of oocyte maturation is characterized by changes in chromosome morphology, as well as dynamic changes in histone modifications. HDACs are chromatin-remodelling proteins that participate in this process by catalysing histone deacetylation. HDACs are co-localized on chromosomes in germinal vesicles oocytes, and following germinal vesicle breakdown (GVBD), they mix with the cytoplasmic contents and cause deacetylation^47^. Deacetylation is essential for normal chromosome condensation and segregation. In support of this concept, conditional knockout or inhibition with TSA results in global histone hyperacetylation, disruption of chromatin configuration and chromosome segregation defects^48^.

Since BOHB is a HDAC inhibitor, we decided to investigate whether exposure of COCs to BOHB would affect histone acetylation levels. After confocal measurement of oocytes from each group, we did not observe any difference in H3K9ac levels between the control oocytes (52.76 ± 2.23; N = 61) and the ones treated with BOHB (56.95 ± 2.74; N = 61), P = 0.24 (Fig. 6D and E). Additionally, since hyperacetylation causes chromatin loosening, we measured the relative nuclear area to investigate whether BOHB affects the relative area/organization of the metaphase plate. As illustrated on Fig. 6D, there was no difference in the diameter of the metaphase plate and no noticeable alteration in chromosome organization, with a relative nuclear area of 0.06058 ± 0.003816 (arbitrary units) for the control group and 0.06039 ± 0.003852 for the treated group, P =  0.97 (Fig. 6F). The absence of detrimental effects in our *in vitro* model suggests that either the ketone body BOHB is not toxic to the oocyte or higher concentrations or longer exposure periods are necessary to affect oocyte development/maturation during folliculogenesis. Additionally, at least in porcine oocytes, the nuclear deacetylases are not required for histone deacetylation during meiotic maturation^49^, which is consistent with the absence of an effect of BOHB on oocyte acetylation levels in our system.

### Effect of BOHB on early embryonic development

#### Treatment of parthenogenetic zygotes with β-hydroxybutyrate (BOHB) does not affect preimplantation embryonic development or ATP content

Although the epigenetic effect of ketone bodies during embryo development in mammals is largely unexplored, there are few reports showing a teratogenic effect of β-hydroxybutyrate when applied at a high concentration^50^ (i.e., ~20 mM; levels reached in human diabetic ketoacidosis), as well as other reports showing that they can be used as an energy source during bovine embryonic development^51,52^.

We asked whether BOHB is suitable for addition to the culture medium to promote embryonic development after somatic cell nuclear transfer (SCNT). Initially, we opted to test the toxicity in parthenotes, since, as for cloned embryos, *in vitro* fertilized embryos have the influence of the spermatozoon and it is impossible to determine the exact time of fertilization to initiate the BOHB treatment. In this experiment, since our main goal was to test the toxicity of BOHB, we selected the concentration of 6 mM, which is close to the maximum levels found in bovines suffering from severe clinical ketosis^16^. Additionally, based on the IC_50_ values of the HDACs 1, 3 and 4, which range from 2 to 5.3 mM, the concentration of 6 mM should provide sufficient *in vitro* inhibition^13^.

**Figure 7:**
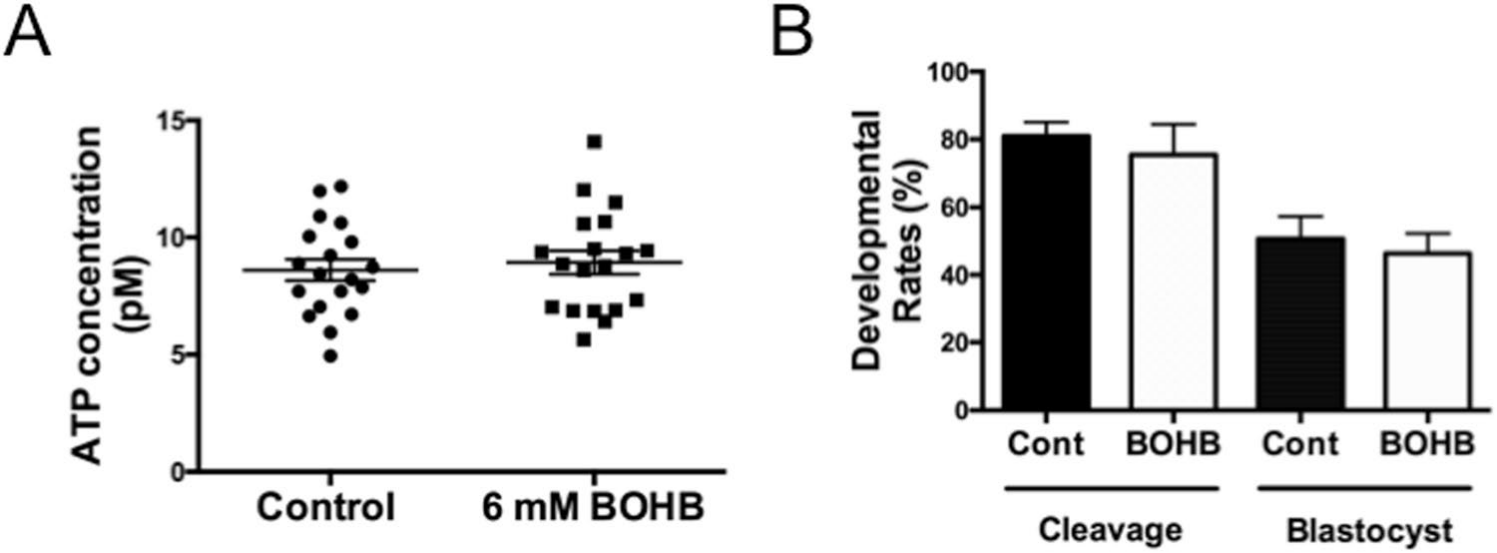
Treatment of parthenogenetic zygotes with β-hydroxybutyrate (BOHB) does not affect preimplantation embryonic development or ATP content. (**A**) Scattered dot plots showing the quantification of ATP content in parthenogenetic zygotes in untreated controls and after treatment with 6 mM of BOHB for 12 h. Each dot represents a pool of 3 parthenogenetic zygotes; lines indicate the means ± s.e.m. (**B**) Cleavage and blastocyst rates of parthenogenetic embryos treated or not treated with 6 mM BOHB. The results are based on 3 independent replicates.

ATP is the currency of the cell, and insults affecting cell physiology tend to affect ATP levels. Additionally, BOHB metabolism generates acetyl-CoA, which is fed into the tricarboxylic acid cycle for oxidation and ATP production^8^. Therefore, we supplemented the embryo culture medium of parthenogenetic zygotes with 6 mM of BOHB and measured their ATP content. We observed similar ATP contents in the control (8.61 ± 0.45 pM) and treated (8.94 ± 0.50 pM) embryos (P = 0.63), suggesting that this metabolite does not affect energy production/consumption in early parthenogenetic zygotes (Fig. 7A).

Further, we cultured parthenogenetic embryos from each group to evaluate any potential harmful effects of BOHB on preimplantation embryonic development. After 3 independent replicates (n = 148), we did not observe prejudicial effects on cleavage (control 80.93 ± 4.22 vs BOHB 75.45 ± ± 8.99; P = 0.38) or blastocyst (control 50.64 ± 6.76 vs BOHB 46.26 ± 5.94; P = 0.56) rates (Fig. 7B), indicating that BOHB is a suitable metabolite to add to the culture media to investigate its epigenetic effect on embryos. The *in vitro* findings may give some clues toward understanding the *in vivo* scenario. As with oocytes, the reduced fertility of dairy cows is also believed to be caused by the production of low-quality embryos. However, our data presented here support the idea that embryos develop normally in the presence of high concentrations of BOHB (i.e., 6 mm BOHB for 12 h), which may represent an adaptation helping bovine embryos survive periods of maternal fasting or ketosis^51,52^.

#### Cloned zygotes treated with β-hydroxybutyrate (BOHB) show elevated levels of histone acetylation

Whereas the treatment of early SCNT zygotes with HDAC inhibitors shortly after activation has been demonstrated to be beneficial for cloned embryo development in mice^53^, the effects of HDACis in livestock species such as cows remain controversial^54–56^. In this context, BOHB arose as an interesting molecule to examine in bovine SCNT because it is an endogenous metabolite with low toxicity at physiological levels and a potent histone deacetylase inhibitor^13^. Moreover, cattle are sensitive to elevated circulating ketone bodies, as evidenced by their detrimental effect on reproductive performance, particularly in lactating dairy cows^12^. Thus, investigating the roles of BOHB could provide insights into ways to improve bovine cloning and ways to ascertain whether this ketone body affects the metabolism and epigenome of the embryo.

**Figure 8:**
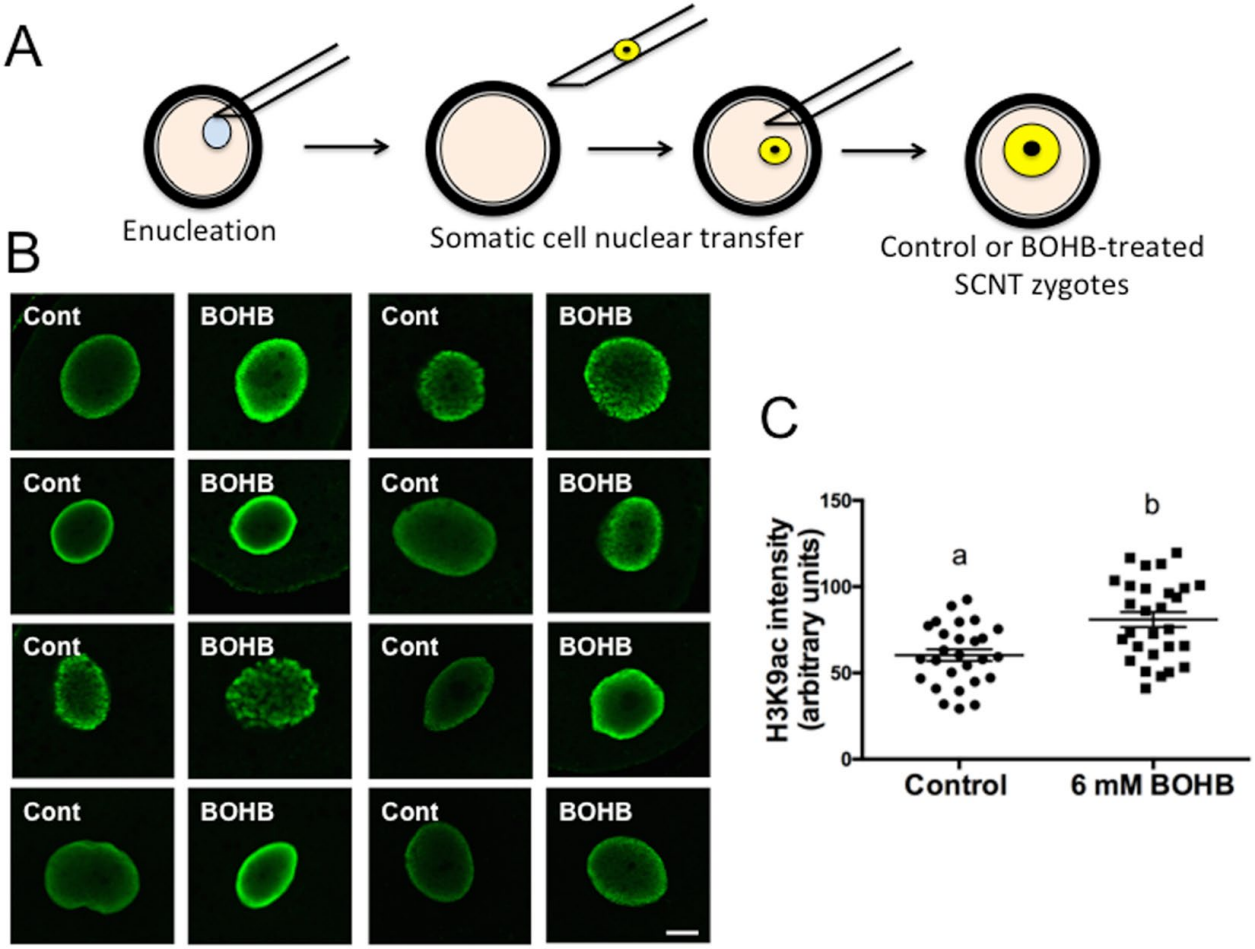
Cloned zygotes treated with β-hydroxybutyrate (BOHB) present elevated levels of histone acetylation. (**A**) Diagram illustrating the SCNT procedure and the treatment of early cloned zygotes with BOHB. (**B**) Immunocytochemical detection of H3K9ac in the nuclei of cloned zygotes treated or not treated with 6 mM of BOHB for 12 h, starting immediately after ionomycin treatment. (**C**) Quantification of H3K9ac signal intensity of SCNT nuclei. Data as presented as the mean ± s.e.m., and different letters indicate a significant difference with respect to the control group (P = 0.0005). All images were taken at the same magnification (40× and 2× digital zoom) and at the same laser power, thereby enabling direct comparison of signal intensities. The scale bar corresponds to 10 μm. Cont: control zygotes; BOHB: zygotes treated with 6 mM BOHB.

Due to a massive wave of epigenetic reprogramming involving chromatin-modifying enzyme activity, early embryos are particularly susceptible to alterations in the culture environment during the first few hours after activation^57–59^, suggesting that this reprogramming process is prone to be affected by HDAC inhibition. In this context, we decided to carry out an experiment exposing early cloned zygotes to high BOHB concentrations (i.e., 6 mM BOHB). Because BOHB inhibits class I and class IIa HDACs with IC_50_ values of 2–5.3 mM, treatment with 6 mM should provide significant inhibition *in vitro*^13^. Additionally, under certain pathological circumstances such as severe clinical ketosis, the concentration of BOHB in the bovine bloodstream is elevated (>3 mM) and can reach ~6–8 mM^15,16^. To investigate whether high levels of BOHB inhibit HDACs in bovine embryos, we treated the cloned zygotes (starting immediately after activation) for 12 h and investigated the levels of H3K9ac at the end of treatment (Fig. 8A). We observed higher levels of histone acetylation in zygotes cultured with BOHB than in control zygotes cultured in standard medium, demonstrating that an endogenous metabolite can affect the global acetylation levels of early-stage embryos. As evaluated by immunostaining analysis (Fig. 8B), the treatment increased the H3K9ac levels of BOHB zygotes (80.97 ± 4.39; N = 28) approximately 1.34-fold (P = 0.0005) compared with the levels in the control group (60.30 ± 3.40; N = 27) (Fig. 8C).

**Figure 9:**
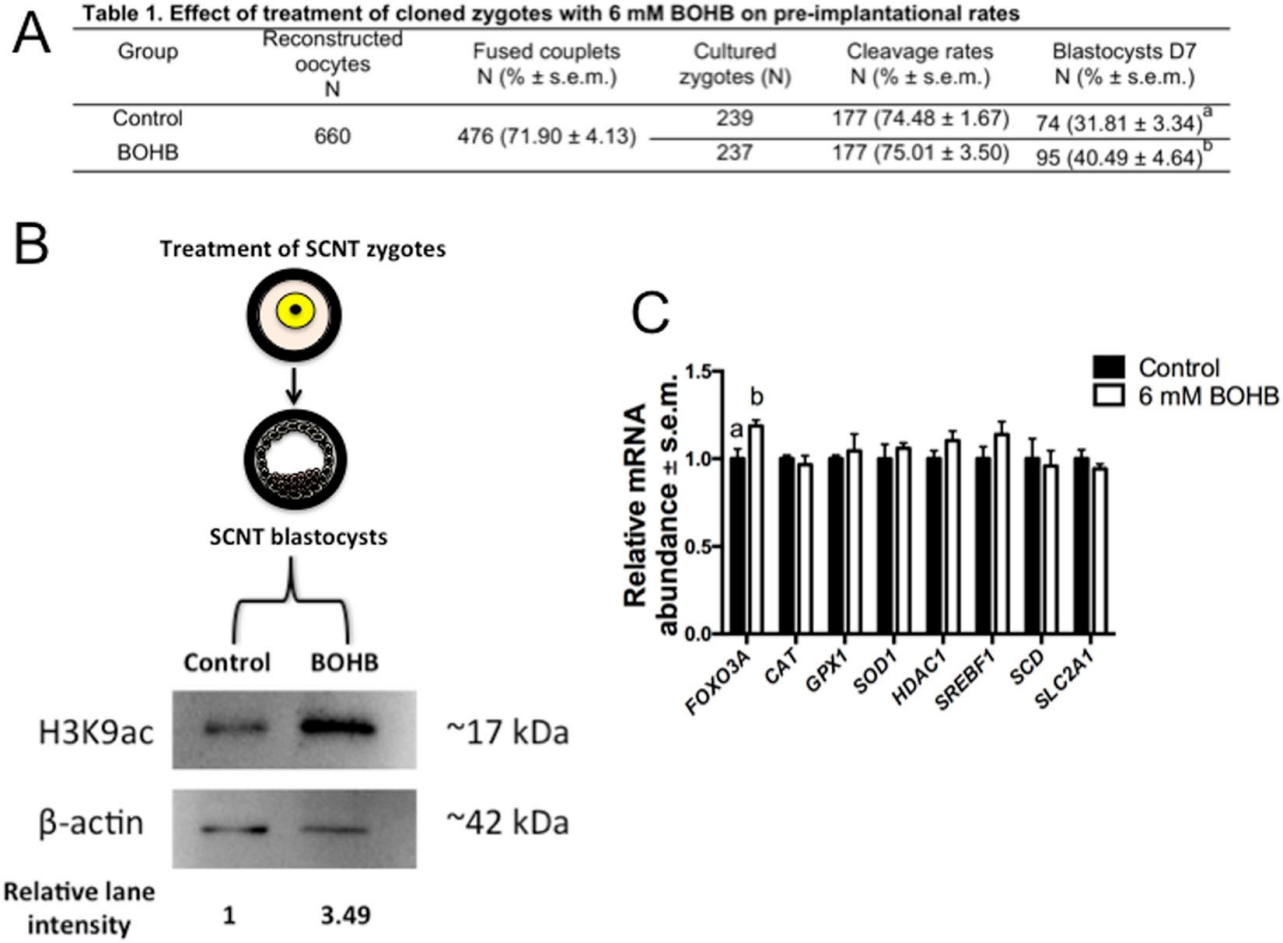
Treatment of SCNT zygotes with β-hydroxybutyrate (BOHB) increased blastocyst development, H3K9ac levels and *FOXO3A* expression. (**A**) Table of SCNT data presenting fusion, cleavage and blastocyst rates. (**B**) Diagram showing the SCNT zygotes, the SCNT blastocysts originating from them, and the immunoblots depicting the H3K9ac levels of day 7 SCNT blastocysts treated or not treated with BOHB at the zygote stage. Relative lane intensity was calculated using the ratio of H3K9ac to ACTB. (**C**) Relative mRNA abundance in SCNT blastocysts from control and BOHB-treated groups collected on day 7 after artificial activation. Data are presented as fold change in each transcript in relation to the control group, and values are shown as the mean ± s.e.m. Gene expression data from 3 biological replicates. Different letters indicate a significant difference with respect to the control group (P < 0.05). Table notes: Fusion rate: number of fused couplets/number of reconstructed oocytes. Cleavage rate: number of cleaved embryos/number of fused couplets. Blastocyst rate: number of blastocysts/number of fused couplets. Values with different superscripts (a, b) within the same column are significantly different (P = 0.0376). The western blot images were cropped for illustrative purposes. The full gels/blots are presented in Supplemental Fig. 9.

HDAC inhibition causes chromatin unfolding by neutralizing the positive charges of histones after acetylation of lysine residues, loosening the chromatin and allowing transcription factors to access their binding sites^60^. During the nuclear reprogramming process, there occurs a well-orchestrated series of events, such as rapid exchange of proteins between the nucleus and the cytoplasm, decondensing of chromatin and a dramatic increase in the nuclear volume^61^, that are believed to improve the development of cloned embryos. Therefore, to test this hypothesis, we performed 7 replicates of SCNT (Fig. 9A, Table 1) and observed a significant (P = 0.0376) improvement in cloned embryo production in the treated group (40.49 ± 4.64%) compared with the untreated control group (31.81 ± 3.34%).

Our observations demonstrated that BOHB affected at least one epigenetic mark (i.e., H3K9ac) in early cloned zygotes and improved preimplantational development. Since histone hyperacetylation is associated with a state that is more permissive to transcription factors due to chromatin loosening^57,60^, the treatment with BOHB may have facilitated the nuclear reprogramming process. Additionally, since epigenetic marks are intertwined^58^, we cannot rule out the hypothesis that other epigenetic modifications (DNA methylation, DNA hydroxymethylation, and histone methylation) were concomitantly altered by our treatment and contributed to the phenotype observed. Additionally, HDAC inhibitors target other pathways essential for early embryo development, such as autophagy, DNA damage repair, stress resistance and metabolic regulation; these may act synergistically to enhance embryonic development, and this possibility warrants further investigation^7^.

#### Blastocysts generated from SCNT zygotes treated with BOHB present elevated levels of H3K9ac and FOXO3A expression

There are critical windows during embryonic development, often coincident with periods of rapid cell division and intense chromatin remodelling, during which an environmental stimulus or insult may have long-lasting consequences for the developing embryo^57^. Early embryonic development is one of these periods, as mentioned above, due to massive epigenetic reprogramming mediated by chromatin-modifying enzymes^58^. That said, molecules affecting these enzymes (e.g., BOHB, which targets HDACs) are presumably more likely to affect the epigenome during this period than at other times. We sought to investigate whether the previous exposure of SCNT zygotes to a “ketotic” microenvironment (6 mM BOHB in SOF embryo culture medium) would affect the H3K9ac levels in cloned blastocysts and consequently alter gene expression and embryonic development. Surprisingly, as observed in the immunoblot (Fig. 9B), the culture of early zygotes in media containing high levels of BOHB led to an increase (~3.49-fold) in the level of H3K9ac in SCNT blastocysts.

These data are intriguing and raise several questions such as the following: Since at least 7 rounds of cell division and extensive chromatin remodelling occur during preimplantation development, how does the brief exposure of zygotes to BOHB increase the H3K9ac levels at the blastocyst stage? It is well known that histone acetylation has a dynamic turnover, and this posttranslational modification is less stable than other epigenetic modifications (e.g., DNA methylation), being less likely to be a carrier of epigenetic information^62^. Nonetheless, there is a report showing that brief exposure of mouse embryos to the HDACi valproic acid alters the acetylation levels of some genes and that this modification is maintained until the morula stage^63^.

Additionally, there are some models explaining the mechanisms by which histone modifications may be maintained in daughter cells after multiple successive cell cycles^64^. These models provide reasonable mechanisms to explain how brief embryonic exposure to BOHB could have increased H3K9ac levels that last until the blastocyst stage. In accordance with our findings, there are observations that high-yielding cows suffering NEB have carryover effects on fertility some months later^3^. This suggests a mechanism by which episodes of ketosis might leave sequelae on the oocyte’s epigenome during oogenesis^43^. Since the composition of oviductal fluid has been shown to resemble that of blood serum, we speculate that BOHB might affect the epigenome of the developing embryo *in vivo*^2^. Recently, compelling evidence has emerged showing that the maternal environment (i.e., diet and stressors) can also induce pertinent changes in the offspring’s metabolism^65^. If these epigenetic marks are inherited during early embryonic development, they might affect the health of the animals even in adulthood. This process, commonly referred as Developmental Origins of Health and Disease (DOHaD), is attracting growing interest in livestock science^65–67^.

Changes in mouse histone acetylation caused by BOHB infusion altered gene expression and promoted protective responses against oxidative stress mediated by the transcription factor *FOXO3A*^13^. Since BOHB inhibits the HDACs and these enzymes repress *FOXO3A* transcription, ketosis may result in transcription of the enzymes of the antioxidant pathways.

Since we observed increased H3K9ac levels in cloned blastocysts treated with BOHB, and histone acetylation is associated with gene expression, we carried out qRT-PCR to analyse the relative expression of genes related to oxidative stress response (*FOXO3A*, *SOD1*, *CAT* and *GPX1*), metabolism (*SREBF1*, *SCD*, *SLC2A1*) and the epigenome (*HDAC1*).

Among all genes investigated, *FOXO3A* was the only one showing higher expression in the BOHB-treated group than in the control group (Fig. 9C). Curiously, *FOXO3A* is the mammalian orthologue of DAF-16 that regulates life span in *Caenorhabditis elegans*^68^ and was demonstrated recently to be necessary for life extension in mice^69^. Even in humans, a particular allele of *FOXO3A* is associated with extreme longevity^70^. Our findings are surprising and demonstrate that a metabolite commonly circulating in cows can affect an epigenetic mark in early zygotes, which persists at least until the blastocyst stage. We speculate that this modification can somehow influence/program the embryos to cope with additional stresses experienced later in life.

## Conclusion

The findings of this study show that the epigenome and metabolism are intimately connected and that it is possible to modify gene expression in cells and embryos, as well as one epigenetic mark in cloned embryos, using an endogenous metabolite during *in vitro* culture. More interesting, we observed that the treatment of embryos with BOHB in the early zygote stage induced an increment in H3K9ac that persisted at least until the blastocyst stage. These findings suggest that the initial hours of embryonic development are important and that the embryo is prone to metabolic and epigenomic alterations depending on the *in vitro* culture environment they face. Our data also reinforce the idea that this ketone body affects somatic cells and embryos; they responded to such an insult by changing their gene expression or reprogramming their metabolism to cope with this altered environment.

Regarding future perspectives, we envision BOHB as a promising molecule because (1) it possesses free radical scavenging activity^8,13^; (2) it might be utilized to mimic calorie restriction *in vitro*^24,71^; (3) it can generate a model to understand the effects of conceiving under starvation in mammals^59,72^; (4) it has anti-diabetic effects^11^, and there is extensive documentation showing that offspring produced by ARTs are more prone to develop insulin resistance than their naturally conceived counterparts are^73,74^; and (5) it can be used in veterinary science as a tool to understand the effects of circulating ketone bodies on dairy cows gametes and embryos^43^, among other topics.

## Methods

All chemicals and reagents used were purchased from Sigma-Aldrich Chemical Company (St. Louis, MO, USA) unless otherwise stated. *In vitro* experimental procedures were carried out in humidified incubators maintained at 38.5 °C in air with 5% CO_2_. The present study was approved by the ethical committee for the use of animals of the School of Veterinary Medicine and Animal Science of the University of São Paulo, protocol number 2546/2012, and complies with the ethical principles of animal research. We adopted the International Guiding Principles for Biomedical Research Involving Animals (Society for the Study of Reproduction) as well.

### Bovine fibroblast isolation and culture

The bovine fibroblast cell line was obtained from a 14-month-old crossbred (Gir x Holstein; *Bos indicus* x *Bos taurus*) heifer. Briefly, a skin biopsy (1 cm^2^) was minced into small pieces with a scalpel blade and digested with 0.1% (w/v) collagenase for 3 h at 38.5 °C. The cell suspension was centrifuged at 300 × *g* for 5 min, and the cell pellet was resuspended in α-MEM (GIBCO BRL, Grand Island, NY, USA) supplemented with 10% (v/v) foetal calf serum (FCS) and 50 μg/ml gentamicin sulfate. The cells were plated in 35-mm Petri dishes and maintained for 6 days in an incubator to establish the primary culture. Thereafter, cells were cultured for an additional two passages; detached from the plate using Tryple Express (GIBCO BRL); resuspended in α-MEM medium supplemented with 10% FCS, 10% (v/v) dimethyl sulfoxide (DMSO), and 50 μg/mL gentamicin sulfate; placed in cryotubes; and stored in liquid nitrogen until use. Regarding the MDBK cells, they were purchased from ATCC, MDBK (NBL-1) (ATCC CCL-22) and cultured as described above for fibroblasts.

### Cell treatment with BOHB for western blotting analysis

A vial of cells (fibroblasts or MDBK) was thawed, and the cells were plated at a density of 5 × 10^4^ cells per 35-mm Petri dish in Dulbecco’s modified eagle’s medium without glucose (DMEM; GIBCO BRL, cat. # 11966-025) supplemented with 10% (v/v) FCS, 2.2 mM glucose and 50 μg/mL gentamicin sulfate. At 24 h after plating, the cell culture medium was replaced with fresh medium containing 0, 2 or 6 mM BOHB, and the cells were cultured for 8 h. Additionally, we treated cells with 1 μM of TSA as positive control for histone hyperacetylation. After treatment, the cells were trypsinized and the histones were acid extracted for western blotting analysis.

### Cell treatment with BOHB for gene expression analysis

Fibroblast cells were cultured as described above, except they were plated at a density of 2.5 × 10^4^ cells per 60-mm Petri dish, and the medium was supplemented with 0 or 2 mM of BOHB. The medium was replenished every 24 h for 4 days, as well as 6 h before the end of treatment. In this case, the rationale was to expose cells to BOHB for a longer period to mimic the *in vivo* conditions in cows with NEB, where ketone body concentrations remain high for several days.

### Cell culture in an acidic extracellular environment

Fibroblast cells were plated at a density of 5 × 10^4^ cells per 35-mm Petri dish in Dulbecco’s modified Eagle’s medium (DMEM; GIBCO BRL, Grand Island, NY, USA, cat. # 11965-092) supplemented with 10% (v/v) and 50 μg/ml gentamicin sulfate and grown for 48 h. Acidic culture medium (pH 6.5) was prepared by adding drops of 12N HCl to DMEM as described previously^23^. Briefly, drops of HCl were slowly added to DMEM medium while stirring. To titrate the pH, an electrode connected to a pH meter was immersed in the beaker containing HCl-acidified DMEM, and the pH decrease was monitored in real time. When the medium reached a pH of 6.5 and the pH meter was stabilized, the medium was sterile filtered. Subsequently, cells were subjected to each condition (regular or HCl-acidified DMEM) for 4 h^22^.

### Gene expression analysis in fibroblasts, cumulus cells and cloned blastocysts

RNA was extracted from fibroblasts, cumulus cells and cloned blastocysts using TRIzol reagent (Invitrogen, Carlsbad, CA, USA) according to the manufacturer’s recommendations, with a few modifications. In brief, a mix containing 1 mL of TRIzol reagent and 20 μg/mL of linear acrylamide (Ambion Inc., Austin, TX, USA, cat. # AM9520) was added to each sample. The extracted RNA was directly dissolved in ultra-pure water and treated with 1 U of DNase I solution (Invitrogen, cat. # 18068015) for genomic DNA degradation, as suggested by the manufacturer. Then, RNA was immediately reverse transcribed into cDNA using the High-Capacity RNA-to-cDNA Kit (Life Tech cat. # 4387406) according to the manufacturer’s protocol and stored at −20 °C until use.

The target genes of interest are listed in Supplemental Table 1, and the reference genes *RPL15, GUSB* and *PPIA* were used as described previously^75^. The primers used for real-time reverse transcription PCR (RT-PCR) were designed using the software Primer Express v3.1 (Applied Biosystems) or Primer-BLAST (NCBI) based upon sequences available in GenBank. Relative quantification of gene-specific mRNA transcripts was performed in 20-μl reactions containing 0.2 mM of each primer plus 1× Power SYBR Green PCR Master Mix (Life Tech cat. # 4367659) and 2 μl of 8-fold diluted cDNA. All gene-specific cDNAs amplified for a particular sample were run in duplicate on the same qRT-PCR plate. A non-template control containing 2 μl of ultra-pure water instead of cDNA was always run in parallel with samples. The following cycling conditions were applied for amplification: initial denaturation at 95 °C for 10 min, followed by 40 cycles consisting of 95 °C for 15 sec and 60 °C for 1 min. The SYBR Green fluorescence was read at the end of each extension step (60 °C). Pilot experiments using five different concentrations of cDNA (spanning a 60-fold range) were run to select the real-time qRT-PCR conditions. The amplification of a single PCR product was confirmed by analysis of the melting curves. The relative abundance of the transcripts was determined using the comparative CT method (ΔΔCT) as described previously^76^. Gene expression data from three biological replicates for blastocysts (pools of 8 blastocysts per group), five biological replicates for fibroblasts and six biological replicates for cumulus cells are presented. To calculate relative expression, the target genes were normalized to the geometric mean of the three most stable reference genes, *GUSB*, *PPIA* and *RLP15*, as described previously^75^.

### Acid extraction of histones from cells

Histones were extracted using the EpiQuick Total Histone Extraction Kit (Epigentek, Farmingdale, NY, USA, cat. # OP-0006) following the manufacturer’s instructions with few modifications. Briefly, harvested cells were pelleted by centrifugation at 300 × *g* for 5 min at 4 °C. Then, the cells were incubated in 200 μL of pre-lysis buffer, and centrifuged at 9,500 × *g* for 1 min at 4 °C. The supernatant was removed, and the pellet was resuspended in 60 μL of lysis buffer and incubated for 30 min at 4 °C. Samples were centrifuged at 13,500 × *g* for 5 min at 4 °C. The supernatant, containing acid-soluble proteins, was transferred into a new vial and neutralized with 0.3 volumes of balance buffer supplemented with DTT. The proteins were quantified by Qubit 2.0 (Life Technologies) and stored in aliquots at −20 °C.

### Coomassie staining

One microgram of acid-extracted histones were mixed with 4× Laemmli buffer (Bio Rad Laboratories, Hercules, CA, USA cat. #161-0747), denatured at 98 °C for 5 min, and loaded into an acrylamide gel. Proteins were fractionated by size on a 4%–15% SDS-PAGE gel run at a constant 100 V for 160 min. The gel was washed 3 times in ultra-pure water and stained with SimplyBlue SafeStain (Thermo Fisher, cat. # LC6060) for 1 h at RT. Next, the gel was washed for 1 h in ultra-pure water to remove the background. The gel image was captured on a ChemiDoc MP Imaging System (Bio-Rad), and enrichment of histones was confirmed.

### Western Blotting for quantification of H3K9ac levels in somatic cells

To measure the relative H3K9ac levels, 0.5 μg of histones were prepared and run as described above. Next, the proteins were electroblotted onto PVDF membranes (Bio-Rad) at a constant 30 V for 70 min using a wet transfer system (Bio Rad). The membranes were blocked with 5% BSA in Tris-buffered saline (TBS) + 0.1% of TWEEN (TBS-T) for 1 h at room temperature. The membranes were incubated overnight at 4 °C under agitation with the primary antibody anti-H3K9ac (Sigma, cat # H9286) diluted 1:5,000 in TBS-T + 1% BSA solution. Subsequently, the membranes were washed 3 times with TBS-T for 5 min each and incubated for 1 h with peroxidase-conjugated anti-rabbit secondary antibody (Sigma, cat # A0545**)** diluted 1:5,000 in TBS-T + 1% BSA solution. The membranes were incubated for 1 min with Clarity Western ECL Substrate (Bio Rad cat. # 170-5060), and images were captured using the ChemiDoc MP Imaging System (Bio-Rad). Images were analysed and bands were quantified using the software Image Lab 5.1 (Bio-Rad). The abundance of H3K9ac was calculated in relation to the loading control histone H3. Membranes were incubated overnight at 4 °C under agitation with anti-histone H3 antibody (Sigma, cat. # H0164) diluted 1:10,000 in TBS-T + 1% BSA solution, then washed and developed as described above.

### Western blotting of proteins from cloned embryos

The organic phase of TRIzol reagent (Invitrogen, Carlsbad, CA, USA) remnant from the gene expression experiment was used to extract the proteins and analyse the H3K9ac levels in cloned blastocysts. We pooled embryos from 3 replicates and extracted the total protein. Briefly, after removal of the aqueous phase using chloroform, the proteins were precipitated from the organic phase by adding 1 mL of isopropanol, incubating the mix at 10 min at RT, and centrifuging at 10,000 × *g* for 10 min at 4 °C. The pellet was washed with 1 mL of 0.3 M guanidine in 95% ethanol, kept for 20 min at RT and centrifuged at 7,500 × *g* for 5 min at 4 °C. Next, the pellet was washed in 100% ethanol, kept for 20 min at RT and centrifuged at 7,500 × *g* for 5 min at 4 °C. The pellet was air dried, resuspended in 1% SDS and used for WB as described above. The only exception is that we used the monoclonal antibody anti-β-actin-peroxidase (Sigma, cat # A3854) diluted 1:100,000 in TBS-T + 1% BSA solution to normalize the H3K9ac in blastocysts.

### Immunostaining of H3K9ac in cloned zygotes and oocytes

Immunostaining was performed as described previously^55^, with few modifications. Oocytes or cloned zygotes were fixed in 3.7% paraformaldehyde (PFA) in PBS for 12 min, washed three times with PBS containing 10 mg/mL BSA (PBS/BSA), and permeabilized with 1% Triton X-100 for 20 min. Then, the samples were washed with PBS/BSA and blocked in PBS + 5% BSA solution for 1 h at RT. Next, the samples were incubated overnight at 4 °C with the primary antibody anti-H3K9ac (1:1,000; Sigma, cat. # H9286) diluted in PBS/BSA. After extensive washing with PBS/BSA, the samples were incubated with the secondary antibody Alexa Fluor 488-conjugated goat anti-rabbit IgG (Life Tech, cat. # A11008) or Alexa Fluor 568-conjugated goat anti-rabbit IgG (Life Tech, cat. # A11036) diluted in PBS/BSA 1:1,500 for 1 h at room temperature. The negative control was obtained by substituting the primary antibody for a rabbit polyclonal IgG isotype control (ABCAM, Cambridge, MA, USA, cat # AB27478). The oocytes and cloned zygotes were then mounted on a glass slide in Prolong Gold Antifade Mountant (Life Tech, cat. # P36935).

Images were captured using a confocal microscope (TCS-SP5 AOBS; Leica, Soims, Germany), and the same settings were used for all images within the same experiment (i.e., analysis of oocytes or cloned zygotes). All samples for a given experiment were processed for immunostaining together, and all images were taken at the same laser power, thereby enabling direct comparison of signal intensities.

The pixel intensity of the images was analysed using the software ImageJ (National Institutes of Health, Bethesda, MD, USA). In this case, over 25 images of individual cloned zygotes per group and over 60 images of oocytes per group were analysed to measure H3K9ac levels. Individual nuclei were outlined, and the average signal intensity was calculated. Intensity refers to the degree of brightness of the coloured pixels (in greyscale: 0–255; 0  =  black and 255 = white), where brighter intensity indicates greater immunostaining reactivity.

### Measurement of ATP content in parthenogenetic early zygotes

The ATP content of 19 pools (3 zygotes each pool) per group (Control or Treated with 6 mM of BOHB, as described below) was measured using the ApoSENSOR™ ATP Cell Viability Bioluminescence Assay Kit (BioVision, Milpitas, CA, USA, cat. # k-254) according to the manufacturer’s instructions. Briefly, a mix containing 10 μl of “ATP monitoring enzyme” and 90 μl of “Nucleotide releasing buffer” were prepared for each sample and added to a 96-well plate. The plate was read on a BMG Labtech Fluorstar Optima microplate reader (BMG Labtech, Ortenberg, Germany) to obtain the background luminescence values. Next, 50 μl of “Nucleotide releasing buffer” was mixed gently into each microtube containing the zygotes, and the contents were transferred to a 96-well plate. Samples were incubated for 2 min at RT and read to obtain the luminescence values. The background luminescence was subtracted from all readings. In parallel, a five-point ATP standard curve (0.15, 0.075, 0.0375, 0.01875, and 0.009375 μM) was generated and used to estimate the ATP concentration in each sample.

### Oocyte recovery and *in vitro* maturation

Ovaries were collected from a local slaughterhouse and transported to the laboratory in an insulated container filled with saline solution (0.9% NaCl) at ~28 °C. The ovaries were washed several times and placed in saline solution for oocyte aspiration. Oocytes were aspirated from 2 to 8 mm antral follicles using an 18 G needle connected to a syringe. Cumulus-oocyte complexes (COCs) containing compact cumulus cell layers and homogeneous cytoplasm were selected and matured in groups of 15–20 COCs in 90 μl of M199 with Earle’s salts, L-glutamine, 2.2 g/L sodium bicarbonate (GIBCO BRL, Grand Island, NY, USA cat. # 11150-059), 1 mg/mL FSH (Folltropin; Ourofino Saúde Animal, Cravinhos, Brazil), 50 mg/mL hCG (Vetecor; Ourofino Saúde Animal), 50 μg/mL gentamicin sulfate, 0.2 mM sodium pyruvate, and 10% FBS. *In vitro* maturation (IVM) was performed for 21-23 h in a humidified atmosphere of 5% CO_2_ in air at 38.5 °C.

### Treatment of cumulus-oocyte complexes with BOHB during *in vitro* maturation

Oocytes were matured as described above, except that the IVM media was supplemented or not supplemented with 2 mM of BOHB during IVM^44^. As a positive control for WB in cumulus cells, COCs were maturated in the presence of 100 nM TSA.

### *In vitro* fertilization

Oocytes matured as described above were *in vitro* fertilized with frozen-thawed semen from a single Nellore bull. Capacitated sperm were obtained after Percoll gradient (45% and 90%) separation. The fertilization medium was composed of Tyrode’s lactate stock, 50 μg/mL gentamicin, 22 μg/mL sodium pyruvate, 40 μL/mL PHE (2 mM D-penicillamine, 1 mM hypotaurine and 245 μM epinephrine), 5.5 IU/mL heparin, and 6 mg/mL bovine serum albumin (BSA). Presumptive zygotes were partially denuded after 18 h and cultured in SOF medium in an incubator with a humidified atmosphere of 5% CO_2_ in air at 38.5 °C for 7 days (fertilization is considered day 0). Cleavage and blastocyst rates were evaluated at 48 and 168 h after *in vitro* fertilization, respectively.

### Production of parthenogenetic embryos

After IVM, matured oocytes had their cumulus cells removed by incubation in 2 mg/mL hyaluronidase and gentle pipetting. The denuded oocytes were washed with M199 containing 25 mM HEPES (Gibco, cat. # 12350-039) and artificially activated by incubation with 5 μM ionomycin diluted in M199 supplemented with 1 mg/mL BSA. Next, presumptive zygotes were washed 3 times in M199 + 30 mg/mL of BSA and incubated in SOF containing 2 mM 6-DMAP for 3 h. Then, the presumptive zygotes were extensively washed and cultured in SOF for 7 d in a humidified atmosphere of 5% CO_2_ in air at 38.5 °C. The parthenotes used to measure the ATP contents and to investigate the toxicity of BOHB on embryonic development were treated in exactly the same way as the cloned embryos (described below).

### Somatic cell nuclear transfer

Somatic cell nuclear transfer was performed as described previously^55^, with few modifications. Briefly, a cryotube containing frozen fibroblasts was thawed, after which the cells were washed in α-MEM supplemented with 10% FCS and 50 μg/ml gentamicin sulfate and plated at a density of 5 × 10^4^ cells per 35-mm Petri dish. After 24 h, cells were cultured for another 3 d in cell culture media containing 0.5% FCS to arrest the cell cycle at the G1/G0 phase via serum starvation.

Cumulus-oocyte complexes (COCs) were matured as described above. The oocytes were denuded of cumulus cells by gentle pipetting in 2 mg/mL hyaluronidase. The oocytes with visible first polar bodies (PBs) were enucleated by removing the 1^st^ PB and metaphase-II (MII) plate by gentle aspiration using a 15-mm (internal diameter) glass pipette (ES TransferTip; Eppendorf, Hamburg, Germany). Enucleated oocytes were reconstructed by injection of a single fibroblast into the perivitelline space. The resulting couplet was fused in electrofusion solution (0.28 M mannitol, 0.1 mM MgSO_4_, 0.5 mM HEPES and 0.05% BSA in ultra-pure water) by applying one pulse of alternating current (0.05 kV/cm for 5 s) and two pulses of continuous current (1.75 kV/cm for 45 ms). Successfully fused couplets were artificially activated 26 h after IVM. Artificial activation was performed as described above (see “Production of parthenogenetic embryos”). Cleavage and blastocyst rates were evaluated at 48 and 168 h after activation, respectively. In all SCNT replicates, parthenogenetic embryos were generated in parallel and used as controls.

### Treatment of cloned embryos with BOHB after SCNT

Presumptive zygotes were treated (BOHB group) or not treated (control group) with 6 mM BOHB for 12 h starting immediately after ionomycin incubation. The control group was incubated for 3 h with 6-DMAP, then extensively washed in SOF and cultured for 7 d. The treated group was cultured for 3 h in SOF + 6-DMAP + 6 mM BOHB, extensively washed in SOF and incubated in SOF supplemented with 6 mM BOHB for an extra period of 9 h (totaling 12 h of treatment). Subsequently, treated zygotes were extensively washed in SOF and cultured in a similar manner to control zygotes. In addition, some zygotes were fixed in PBS–PVP supplemented with 4% paraformaldehyde at the end of treatment (12 h after activation) for immunostaining.

### Statistical analysis

Statistical analysis was performed using the SAS System v. 9.3 (SAS/STAT, SAS Institute Inc., Cary, NC, USA) or GraphPad Prism 5 (GraphPad Software, San Diego, California, USA). Data were tested for normality of residuals and homogeneity of variances using the Shapiro-Wilk test and analysed as follows. Immunostaining, ATP contents and gene expression experiments data were analysed by Student’s *t*-test. Western blot data (BOHB and HCl experiments) were analysed by one-way ANOVA followed by Tukey’s post hoc test. Frequency data (oocyte maturation, parthenogenetic embryo development, *in vitro* fertilization and SCNT developmental rates) were analysed using the chi-squared test. Differences with probabilities of P < 0.05 were considered significant. In the text, values are presented as the means ± the standard error of the mean (s.e.m.). All experiments were repeated at least three times unless otherwise stated.

## Electronic supplementary material

Supplementary Information

## Data Availability

The datasets generated during and/or analysed during the current study are available from the corresponding author on reasonable request.
