# Author Correction: Metabolic gene expression and epigenetic effects of the ketone body β-hydroxybutyrate on H3K9ac in bovine cells, oocytes and embryos

**DOI:** 10.1038/s41598-018-35527-9

**Published:** 2018-11-16

**Authors:** Juliano Rodrigues Sangalli, Rafael Vilar Sampaio, Maite del Collado, Juliano Coelho da Silveira, Tiago Henrique Camara De Bem, Felipe Perecin, Lawrence Charles Smith, Flávio Vieira Meirelles

**Affiliations:** 1 University of Sao Paulo, Department of Veterinary Medicine, Faculty of Animal Science and Food Engineering, Pirassununga, Sao Paulo postcode: 13635-900 Brazil; 2 Université de Montréal, Faculté de médecine vétérinaire, Centre de recherche en reproduction et fertilité, St. Hyacinthe, Québec, postcode: H3T 1J4 Canada

Correction to: *Scientific Reports* 10.1038/s41598-018-31822-7, published online 13 September 2018

The Acknowledgements section in this Article is incomplete.

“The authors would like to thank the LMMD members for the help with sample collection and laboratory procedures, critical reading and discussion of the manuscript. The MDBK cells were kindly donated by Dr. Clarice Weis Arns. Juliano Rodrigues Sangalli is supported by São Paulo Research Foundation - FAPESP, grant number 2016/13416-9 and previously by the grants 2013/06673-7 and 2014/21097-5. This work was granted by São Paulo Research Foundation – FAPESP, grant number 2012/50533-2, 2013/08135-2, 2014/21034-3, 2014/22887-0. The funders had no role in study design, data collection and analysis, decision to publish, or preparation of the manuscript.”

should read:

“The authors would like to thank the LMMD members for the help with sample collection and laboratory procedures, critical reading and discussion of the manuscript. The MDBK cells were kindly donated by Dr. Clarice Weis Arns. Juliano Rodrigues Sangalli is supported by São Paulo Research Foundation - FAPESP, grant number 2016/13416-9 and previously by the grants 2013/06673-7 and 2014/21097-5. This work was granted by São Paulo Research Foundation – FAPESP, grant number 2012/50533-2, 2013/08135-2, 2014/21034-3, 2014/22887-0 and 2014/50947-7; CNPq grant number 465539/2014-9 and CAPES. The funders had no role in study design, data collection and analysis, decision to publish, or preparation of the manuscript.”

