# Supplementary material for: Metabolic gene expression and epigenetic effects of the ketone body β-hydroxybutyrate on H3K9ac in bovine cells, oocytes and embryos
Source: Sci Rep. 2018 Sep 13;8:13766. doi: 10.1038/s41598-018-31822-7 (PMC6137158; doi:10.1038/s41598-018-31822-7)
Supplement: Supplementary file 1 — Supplementary Information [file 41598_2018_31822_MOESM1_ESM.pdf]

Title: Metabolic gene expression and epigenetic effects of the ketone body β-hydroxybutyrate on H3K9ac in bovine cells, oocytes and embryos

Authors: Juliano Rodrigues Sangalli*, Rafael Vilar Sampaio, Maite del Collado, Juliano Coelho da Silveira, Tiago Henrique Camara de Bem, Felipe Perecin, Lawrence Charles Smith & Flávio Vieira Meirelles.

*julianors@usp.br

Supplemental table 1. Primer sequences used in real time RT-PCR.

Gene Symbol	Gene name	Accession number	Primer Sequences (5'-3')	Product (bp)	AT (°C)
GUSB	glucuronidase, beta	NM_001083436.1	F: AGAGCGAGTACGGAGCAGATG<br>R: AGCAGGCCTTTCTGGTACTCTTC	85	60
PPIA	Peptidylprolyl isomerase A (cyclophilin A) (PPIA)	NM_178320.2	F: GGTCCTGGCATCTTGTCCAT<br>R: TGCCATCCAACCACTCAGTCT	94	60
RPL15	Ribosomal protein L15	NM_001077866.1	F: CAAACGCCCAGTTCCTAAGG<br>R: TCGAGCAAACTTGAGCTGGTT	94	60
HDAC1	Histone deacetylase 1	NM_001037444.2	F: TTACGACGGGGATGTTGGAA<br>R: GGCTTTGTGAGGGCGATAGA	136	60
SIRT1	Sirtuin 1	NM_001192980.1	F: TTGCAACAGCATCTTGCCTG<br>R: GACATCGAGGAACCACCTGAT	90	60
SIRT3	Sirtuin 3	NM_001206669.1	F: TGGCGTTGTTTCCTCGTTCA<br>R: CTGGAATACGAGGCCAGCG	90	60
PPARA	Peroxisome proliferator activated receptor alpha	NM_001034036	F: CGGAAGGCTACTCCACGTTT<br>R: TCACTGGTAAATTCCTAACACGA	193	60
PPARGC1A	Peroxisome proliferator-activated receptor gamma, coactivator 1 alpha	NM_177945.3	F: TGTGCTCTGTGTCACTGTGG<br>R: GAGCAGCACACTCGATGTCA	120	60
SREBF1	Sterol Regulatory Element Binding Transcription Factor 1	NM_001113302.1	F: TGCTGACCGACATAGAAGACAT<br>R: CGTAGGGCGGGTCGAATAG	81	60
SLC2A1	Solute carrier family 2 (facilitated glucose	NM_174602.2	F: CAAGAGAGTCGCAGACGGAG<br>R: CCTGTCAGCTTCTTGCTGGT	135	60

	transporter), member 1				
SCD	Stearoyl-CoA desaturase (delta-9-desaturase)	NM_173959.4	F: GTGGAGTCACCGAACCTACA<br>R: AAACGTCATTCTGGAACGCCA	86	60
CPT1A	Carnitine palmitoyltransferase 1A	NM_001304989.1	F: TACCGACTCACATCCAGGCG<br>R: CGGAGCAGAGCGGAATCGTA	129	60
FOXO3A	Forkhead box O3	NM_001206083.1	F: GCAGGGAGCGCGATATTG<br>R: CGGGCACCATGAATCTGAA	76	60
SOD1	Superoxide dismutase 1, soluble	NM_174615.2	F: AAGATGAAGAGAGGCATGTTGGA<br>R: GATGGCAACACCGTTTTTGTC	65	60
SOD2	Superoxide dismutase 2, mitochondrial	NM_201527.2	F: TCTGTTGGTGTCCAAGGCTC<br>R: AGCAGGGGGATAAGACCTGT	125	60
GPX1	*Glutathione peroxidase 1*	NM_174076.3	F: CGGGTTCGAGCCCAACT<br>R: GCGCCTTCTCGCCATTC	59	60
CAT	*Catalase*	NM_1035386.2	F: CTATGGCCTCCGCGATCTTT<br>R: CGTGAGGCCAAACCTTGGTA	140	60
SLC2A3	Solute carrier family 2 (facilitated glucose transporter), member 3	NM_174603.3	F: CTGGAAGAGGGGTCTCTGGA<br>R: CAGAGGTGCGGTGACCTTC	101	60

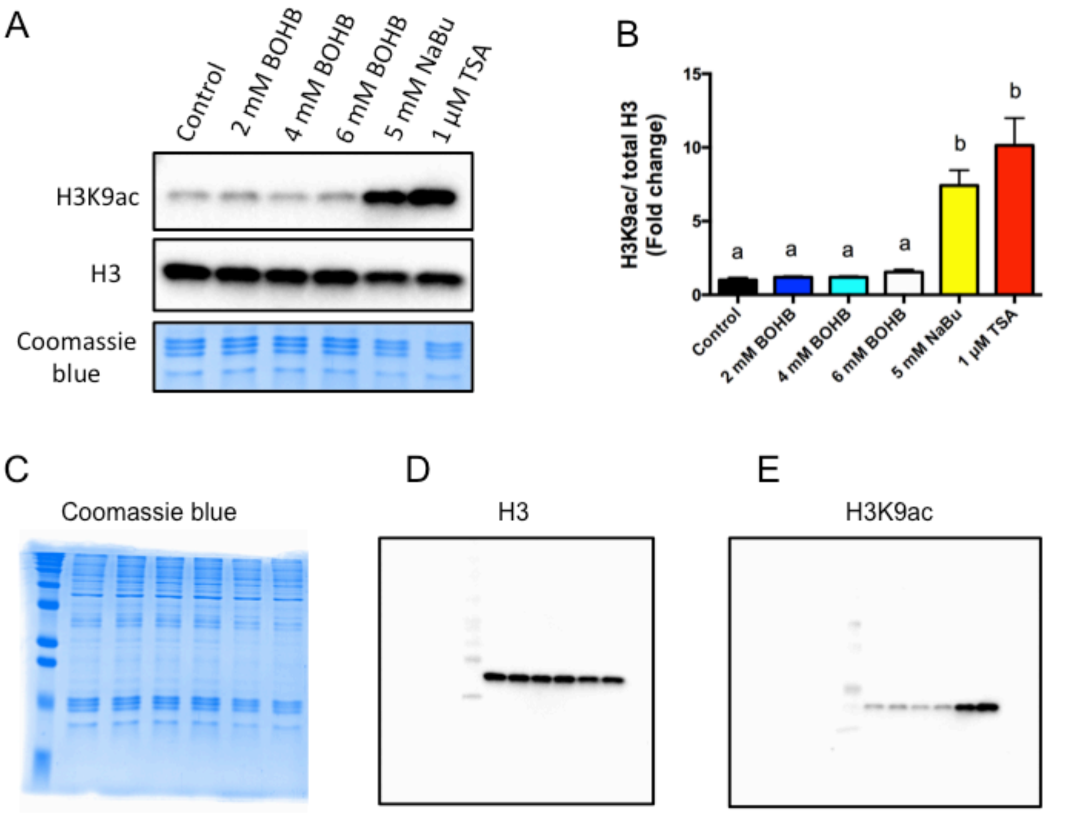

Supplemental Figure 1. (A) Immunoblots and (B) quantification values of H3K9ac in skin fibroblasts treated for 24 h with 0 mM (control), 2 mM, 4 mM or 6 mM BOHB. Additionally, we treated the cells with 5 mM sodium butyrate (NaBu) or 1 µM TSA as positive controls. Data are representative of 3 independent WBs, and H3K9ac levels were calculated in relation to total histone 3 (H3). Data are presented as fold change in relation to the control group and are shown as the mean ± s.e.m. Different letters indicate a significant difference ($P < 0.05$). The western blot images were cropped for illustrative purposes. The full gels/blots are presented below. Supplemental Figure 1 (C) Coomassie blue gel. Supplemental Figure 1 (D) Total histone 3. Supplemental Figure 1 (E) H3K9ac.

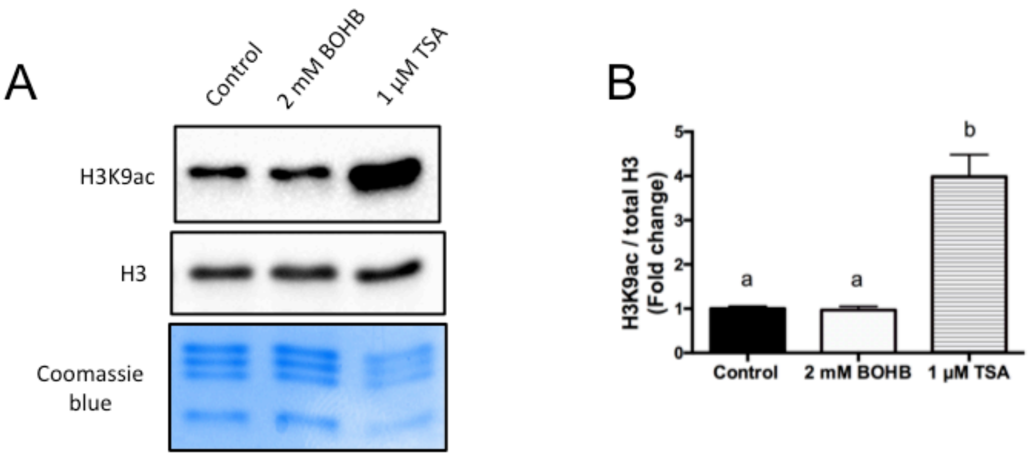

Supplemental Figure 2. (A) Immunoblots for H3K9ac on skin fibroblasts treated with control (0 mM BOHB) or 2 mM BOHB for 96 h and cells treated with 1 μM TSA for 8 h. (B) Quantification of H3K9ac levels on fibroblasts treated for 96 h. Data are representative of three pairs of biological replicates. The H3K9ac levels were calculated in relation to total Histone 3 (H3). Data are presented as fold change in relation to the control group, and the values are shown as the mean ± s.e.m. Different letters indicate statistical difference ($P < 0.05$).

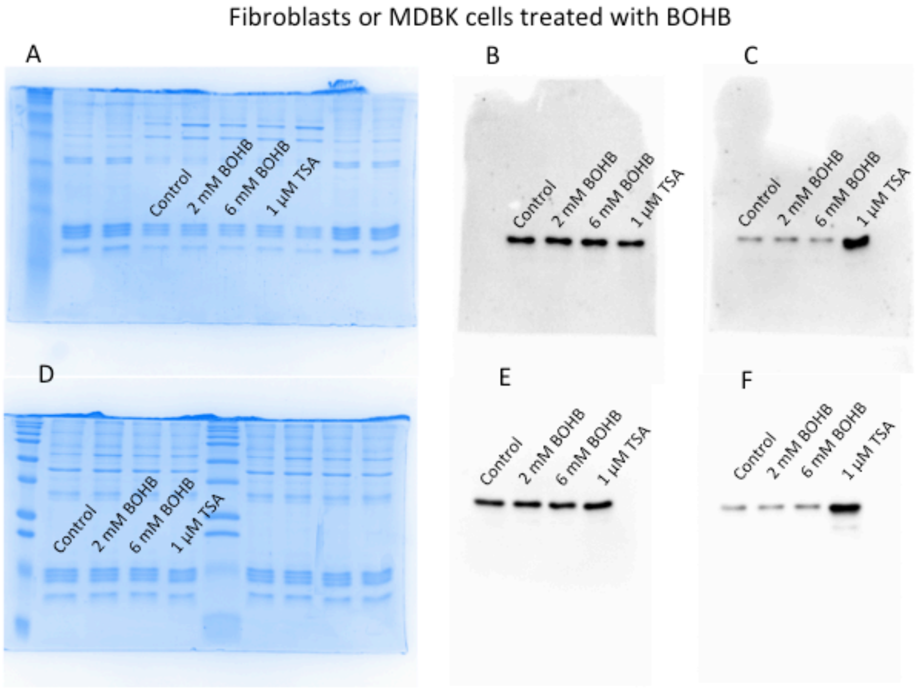

Supplemental Figure 3. Full immunoblots from data presented on Figure 1. (A) Coomassie staining in fibroblasts cells. (B) Immunoblot for total Histone H3 in fibroblasts. (C) Immunoblot for H3K9ac in fibroblasts. (D) Coomassie staining in

MDBK cells. (E) Immunoblot for total Histone H3 in MDBK cells. (F) Immunoblot for H3K9ac in MDBK cells.

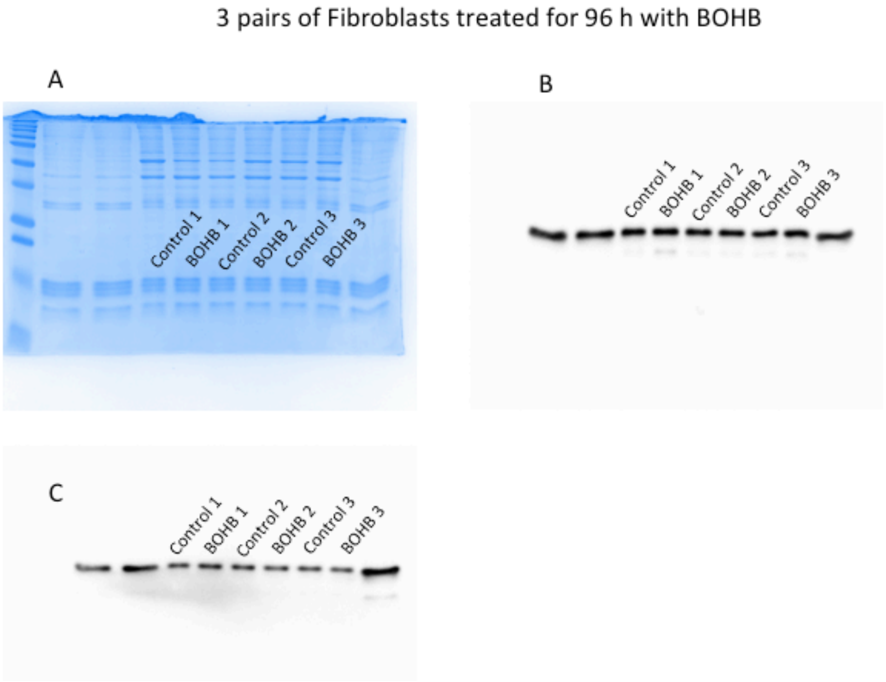

Supplemental Figure 4. Full immunoblots from data presented on Figure 2. (A) Coomassie staining in 3 pairs of fibroblasts. (B) Immunoblot for total Histone H3 in 3 pairs of fibroblasts. (C) Immunoblot for H3K9ac in 3 pairs of fibroblasts.

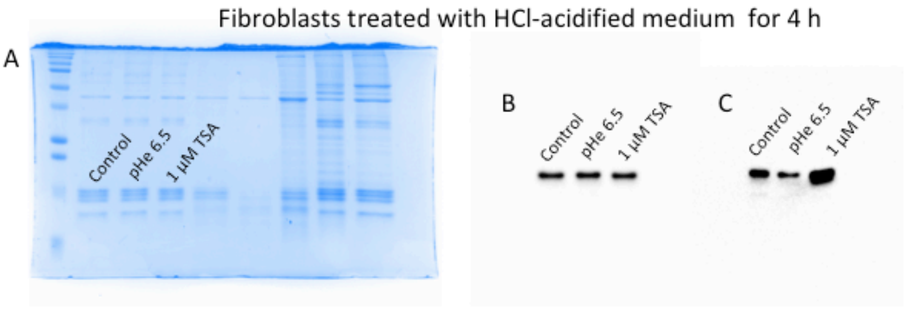

Supplemental Figure 5. Full immunoblots from data presented on Figure 3. (A) Coomassie staining in fibroblasts from control, HCl or Trichostatin A treated groups. (B) Immunoblot for total Histone H3. (C) Immunoblot for H3K9ac.

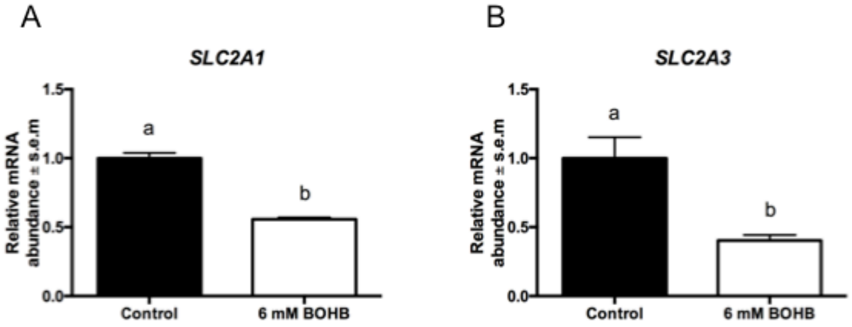

Supplemental Figure 6. Quantification of *SLC2A1* (A) and *SLC2A3* (B) transcripts in bovine skin fibroblasts cultured either with (6 mM) or without (control) BOHB for 12 h. Transcript amounts were normalized utilizing the geometric mean of housekeeping genes (*GUSB*, *PPIA* and *RPL15*). Data are presented as fold change in relation to the control group, and the values are shown as the mean ± s.e.m. Different letters indicate statistical difference (P < 0.05).

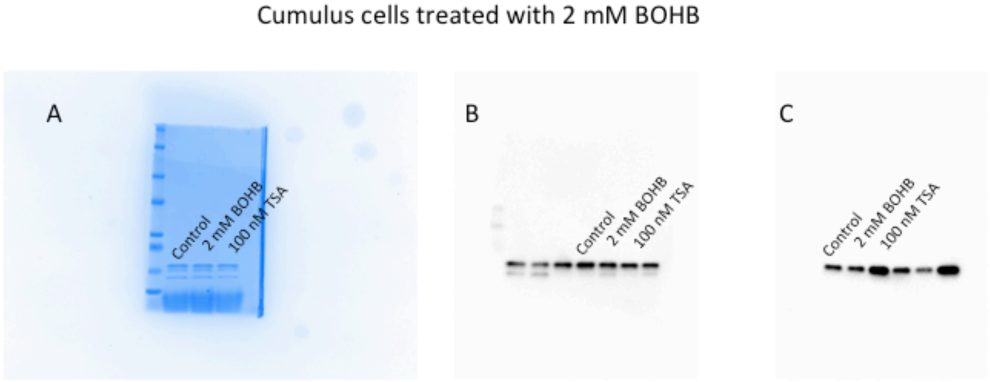

Supplemental Figure 7. Full immunoblots from data presented on Figure 5. (A) Coomassie staining in cumulus cells from control or BOHB treated group (B) Immunoblot for total Histone H3 in cumulus cells from control or BOHB treated group. (C) Immunoblot for H3K9ac in cumulus cells from control or BOHB treated group.

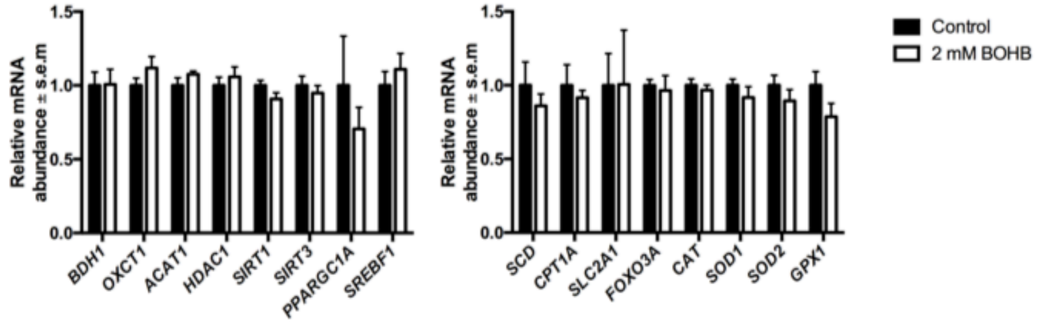

Supplemental Figure 8. Quantification of transcripts in bovine cumulus cells cultured either with (2 mM) or without (control) BOHB for 21-23h h. Transcript quantities were normalized utilizing the geometric mean of housekeeping genes (*GUSB*, *PPIA* and *RPL15*). Data are presented as fold change in relation to the control group, and the values are shown as the mean ± s.e.m.

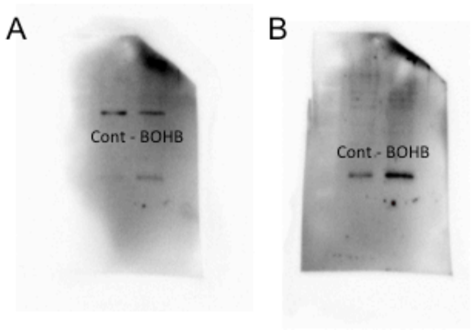

Supplemental Figure 9. Full immunoblots from data presented on Figure 9. (A) Immunoblots depicting β-actin levels on day 7 SCNT blastocysts treated or not with BOHB during the zygote stage. (B) Immunoblots depicting H3K9ac levels on day 7 SCNT blastocysts treated or not with BOHB during the zygote stage.
